# Dried blood sample analysis by antibody array across the total testing process

**DOI:** 10.1038/s41598-021-99911-8

**Published:** 2021-10-15

**Authors:** Kelly Whittaker, Ying-Qing Mao, Yongping Lin, Huihua Zhang, Siwei Zhu, Hannah Peck, Ruo-Pan Huang

**Affiliations:** 1 RayBiotech Life, 3607 Parkway Lane, Peachtree Corners, GA 30092 USA; 2 RayBiotech Guangzhou, 79 Ruihe Road, Huangpu district, Guangzhou, 510630 Guangdong China; 3 Department of Laboratory Medicine, The First Affiliated Hospital of Guangzhou Medical University, Guangzhou, 510000 Guangdong China; 4 South China Biochip Research Center, 79 Ruihe Road, Huangpu District, Guangzhou, 510600 Guangdong China; 5 Affiliated Cancer Hospital and Institute of Guangzhou Medical University, Guangzhou Medical University, Guangzhou, 510095 China; 6 Guangdong Provincial Hospital of Chinese Medicine, Guangzhou, 510120 China; 7 Present Address: Wallace H. Coulter Department of Biomedical Engineering, Georgia Institute of Technology and Emory University, Krone Engineering Biosystems Building, 950 Atlantic Dr. NW, Atlanta, GA 30332 USA

**Keywords:** High-throughput screening, Proteomic analysis, Cytokines, Assay systems

## Abstract

Dried blood samples (DBSs) have many advantages; yet, impediments have limited the clinical utilization of DBSs. We developed a novel volumetric sampling device that collects a precise volume of blood, which overcomes the heterogeneity and hematocrit issues commonly encountered in a traditional DBS card collection as well as allowing for more efficient extraction and processing procedures and thus, more efficient quantitation, by using the entire sample. We also provided a thorough procedure validation using this volumetric DBS collection device with an established quantitative proteomics analysis method, and then analyzed 1000 proteins using this approach in DBSs concomitantly with serum for future consideration of utility in clinical applications. Our data provide a first step in the establishment of a DBS database for the broad application of this sample type for widespread use in clinical proteomic and other analyses applications.

## Introduction

Dried blood sample (DBS) collection, which involves collecting capillary blood through a simple finger prick and applying it to filter paper, was developed over 50 years ago. Its original application was to facilitate newborn heel prick collection^1^. For many years since, the use of DBSs in a clinical setting was predominantly geared towards newborn screening and infection diagnosis in resource-limited locations^2,3^. However, the benefits of DBS collection compared to conventional venous collection have been increasingly appreciated for applications in other clinical fields. These benefits include minimally invasive sample collection, low cost, minimal sample processing requirements, increased stability during both long-term storage and shipping, reduced risk of bacterial contamination or hemolysis, and the ability to obtain high quality samples from remote locations or from patients with limited mobility^4–7^. Recently, application of DBSs in clinical laboratory diagnostics has increased dramatically to include not only newborn screening and infection diagnosis^3,8^ but also toxicokinetic and pharmacokinetic studies^9^, drug development^10^, therapeutic drug monitoring^11^, clinical pharmacology^2^, forensic toxicology^12^, metabolic profiling^2^, environmental control, epidemiological disease surveillance^2,8^, etc. The CDC commented on the quality of DBS collection, noting that “The filter paper blood collection device has achieved the same level of precision and reproducibility that analytical scientists and clinicians have come to expect from standard methods of collecting blood, such as vacuum tubes and capillary pipettes”^13,14^, further lending credibility for the use of DBSs in clinical analysis. Specifically, the current COVID-19 global pandemic has rapidly increased the interest in at-home sample collection and testing, both for convenience and to minimize risk of infection, and one of the easiest at-home blood sampling techniques is DBS collection. Innovative dried blood sample collection tools like the one presented here can be used for monitoring of those who need frequent blood tests as well as for new analyses while avoiding repeatedly coming to a healthcare setting where the risk for infection to not only COVID-19 is increased and to make access to healthcare easier for populations that are located far from hospitals and clinics.

Proteins are responsible for performing many biological functions, and their measurement can inform not only on the normal functioning but also the malfunctioning of proteins that cause disease. Thus, the quantitative analysis of protein biomarkers has emerged as an important tool for clinical applications in disease diagnosis, assessment of disease risk, and monitoring of disease progression and therapeutic effectiveness. Considerable technological advances in proteomic analysis in recent years, specifically in quantitative immunoassay approaches, have enabled cost-effective assays with precise, high-throughput capabilities. While the majority of these assays have been designed for use in serum or plasma^15^, only a few have been validated for DBSs. Martin et al. validated the use of an enzyme-linked immunosorbent assay (ELISA) to quantify adiponectin in a large-scale multi-center trial^16,17^. Commercially available ELISAs have also been evaluated and optimized for the detection of various proteins in DBSs, including CRP^18,19^, alpha 1-acid glycoprotein^19^, IgA^20^, HCV antibodies^21^, biologic exenatide^22^ and an anti-CD20 monoclonal antibody drug^23^. Recent studies have also validated multi-plex immunoassay approaches with DBSs, further increasing their utility for clinical applications by expanding the breadth of analytes detected. Bead-based multi-plex protein arrays^24–27^, reverse-phase peptide arrays^28^, and planar antibody arrays^6^ have been similarly utilized. However, impediments remain to the full realization of DBSs in proteomic analysis.

Two main disadvantages of DBS collection limit its utility for proteomic techniques in clinical applications: (1) the small sample volume, and (2) area bias and homogeneity issues. While the small sample volume consumption can be advantageous (e.g. for pediatric patients from which limited quantities of blood can be collected), it can also pose a significant sensitivity limitation, particularly for non-abundant proteins^29^. Thus, the sample processing, including collection, extraction, and handling, is of paramount importance in DBS assay validation. Similarly, non-uniform blood distribution across a DBS, as well as changes in hematocrit levels, contribute heavily to collection volume uncertainty. The implementation of volumetric sampling devices, wherein a controlled volume of blood is collected and/or used for analysis, greatly reduces uncertainty. These devices also eliminate the need for punching (used in traditional DBS collection), which saves time, reduces processing, and increases throughput, which improves suitability for automation. It also allows for better quantitation and utilization of the entire sample, which provides more accurate data and allows more proteins to be assessed. A number of volumetric sampling devices have been previously developed, and the advantages and drawbacks to such devices have also been discussed previously^30–37^. Another major impediment to DBSs being widely used in the clinic is the lack of validation of many assays and many protein analytes with DBS. One way to establish confidence in DBS data, as well as increase its implementation a regulated bioanalysis environment, is to provide thorough method validation of the DBS method alongside that of serum/plasma. In addition, validation of a large number of proteins—preferably spanning high and low levels of expression—with an approach that has been established clinically previously (e.g., single-plex or multi-plex quantitative immunoassays) will be necessary to establish confidence and provide a step forward for full implementation of DBSs in clinical applications.

In this study, we aim to (1) provide a thorough procedure validation using a volumetric DBS collection device with an established quantitative proteomics analysis method and (2) analyze 1000 proteins using this approach in DBSs and validate measurable proteins for future consideration of utility in clinical applications. To accomplish these aims, we developed a novel volumetric sampling device to collect a precise volume of blood to overcome the heterogeneity and hematocrit issues commonly encountered in a traditional DBS card collection. The added advantage of using the entire sample also allows for more efficient extraction and processing procedures and thus, more efficient quantitation. We then use this volumetric sampling device to validate and optimize a DBS method concomitantly with serum, using a multi-plex immunoassay for 1000 proteins. Our data provides evidence of the applicability of DBSs for widespread use in clinical applications.

## Experimental methods

### Experimental design and statistical rationale

200 normal serum samples from 100 females, aged 30–70 years of age and 100 males, aged 30–70 years of age were obtained from BioreclamationIVT (Washington D.C., USA). The serum samples were stored at − 80 °C until use. 72 normal DBSs, 42 females aged 30–70 years of age and 30 males, aged 30–70 years of age were self-collected using a finger prick and the directions provided after informed consent was supplied. DBSs were stored at − 20 °C after collection prior to analysis. 21 matched DBS, serum, and Dried Serum Spot (DSS) samples were also assessed. Replicate analyses are explained below for each independent assay.

### DBS volumetric sampling device development, collection, drying, and storing

**Figure 1:**
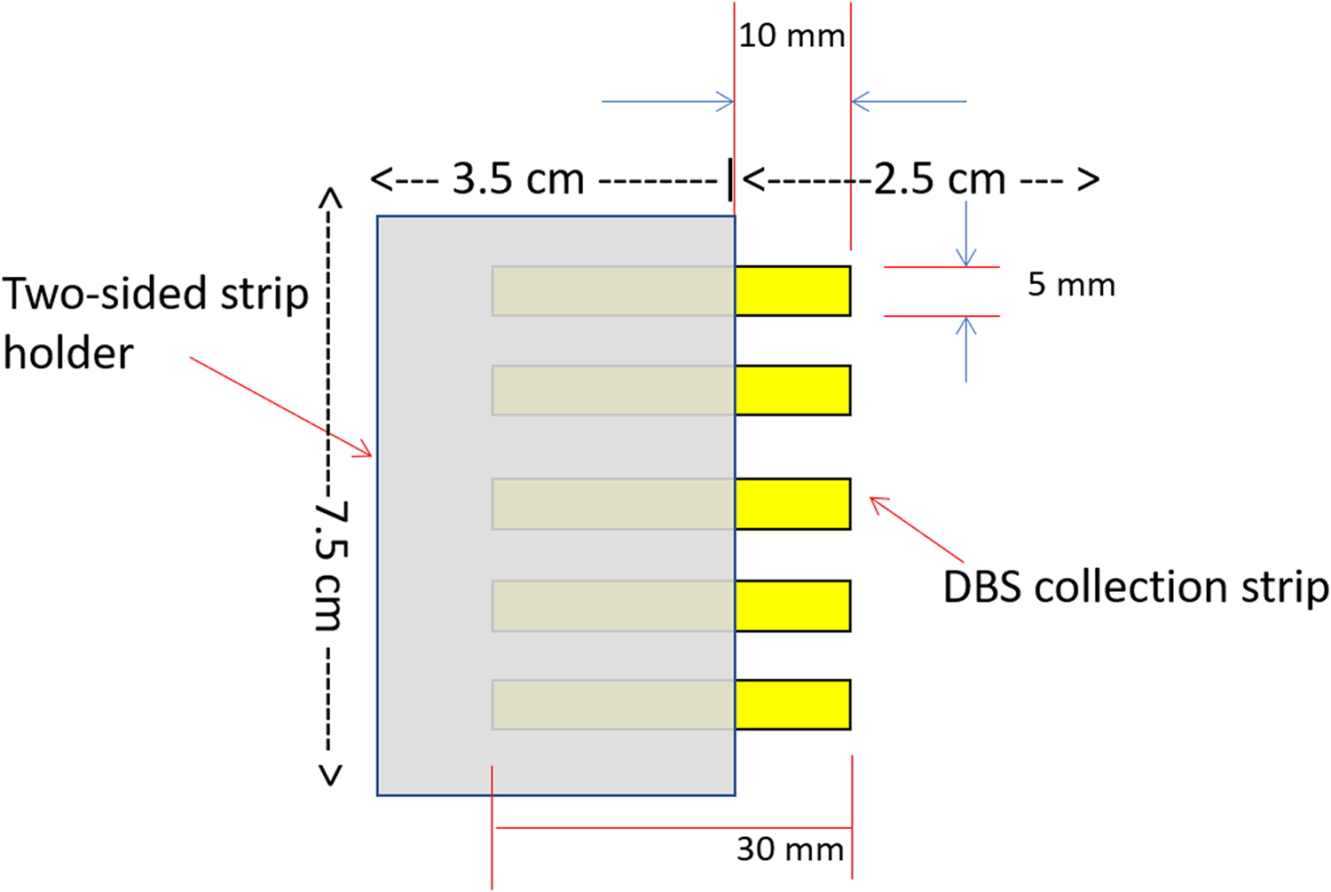
Schematic of volumetric DBS sampling device. The volumetric sampling device consists of 5 30 mm × 5 mm Whatman filter paper strips contained in a holder that allows exposure of a 10 mm × 5 mm section of each strip that collects a 30 μL volume of blood.

The volumetric sampling device consists of a Whatman filter paper strip contained in a 30 mm × 5 mm polyvinyl chloride (PVC) holder that exposes a 10 mm × 5 mm section of filter paper. Five strips are enclosed in a 6 cm × 7.5 cm cardboard 2-sided strip holder that maintains an 8 mm space between strips to prevent contamination (Fig. 1). The volume of blood absorbed by each strip was assessed using an Accuris instruments scale (W3200-120, Accuris Instruments, Edison, USA).

The collection, drying, and storing of DBSs was conducted as follows: (1) The operator washed their hands with warm water, and stimulated blood flow towards the fingertips by massaging and/or shaking the hand. (2) The fingertip was cleaned with an alcohol swab and punctured using a lancet. (3) A large drop of blood was created. Then, the blood collection device was touched to the blood drop and the blood was allowed to fill the device. The device was removed and the finger gently massaged to create another drop of blood. This process was repeated until the blood completely filled the device and white areas disappeared. (4) When the collection device was full, the fingertip was bandaged and the device set aside to dry for 2 h. at room temperature. (5) After the blood was dry, the blood collection device was closed and placed in a sealable biohazard bag with a desiccant pack and closed securely. The package was kept cool and dry until mailing and/or analysis.

### Quantitative immunoassay procedure

DBSs were assessed with an accelerated stability test protocol: samples stored at − 80 °C, 4 °C, ambient temperature (AT), 37 °C, 50 °C, and 60 °C for 0, 1, 4, 8, and 12 weeks. The average protein retention across 14 proteins was determined with a quantitative multi-plex immunoassay according to the manufacturer’s instructions (QAH-SAP-1, Raybiotech Inc, Peachtree Corners, USA). Another quantitative multi-plex immunoassay (QAH-CYT-10, Raybiotech Inc, Peachtree Corners, USA) was used according to the manufacturer’s instructions to determine the optimal elution buffer formula, to determine the optimal sample dilution, and to assess intra- and inter-assay reproducibility. Briefly, samples were incubated with slides pre-printed with the respective capture antibodies. A standard mix, consisting of known amounts of recombinant proteins representing all respective proteins was used to quantify the concentration of every cytokine. Sample incubation was followed by extensive washing to remove any non-bound proteins in the sample matrix. Next, a biotinylated detection antibody cocktail was incubated on the array. After washing away unbound biotinylated detection antibodies, the arrays were incubated with a fluor-conjugated with streptavidin and the fluorescent signals were visualized using a fluorescent scanner (InnoScan 710, Inopsys). Inter- and intra-slide normalization of fluorescent signal intensities was conducted using positive controls, consisting of biotin-labeled IgG, and negative controls, consisting of buffer only. Average signal intensities of quadruplicate spots, minus outliers, were used for all calculations. Outliers were determined as values over 30% above the average signal intensity across the quadruplicate spots. Data below the lower limit of detection (LOD) were discarded^38^. Optimization of the elution buffer was determined by comparing the average protein expression of 40 proteins in 3 samples each of 4 different buffer formulations: 1 × PBS (EB1), 1 × PBS containing 0.1% Tween-20 (EB2), 1 × PBS containing 10% casein and 50% BSA (EB3), and 1 × PBS containing 10% casein, 50% BSA, and 0.1% Tween-20 (EB4). Optimal sample dilution was determined by comparing the average protein expression of 40 proteins between 5 different dilutions of the eluate tested in triplicate: 5 ×, 10 ×, 20 ×, 50 × and 100 ×. Inter- and intra-assay reproducibility was determined in 10 samples, either run at the same time or in separate experiments, respectively. Matched serum, DBS, and DSS samples were assessed using a quantitative multi-plex immunoassay for 200 proteins according to the manufacturer’s instructions (QAH-CAA-4000, Raybiotech Inc, Peachtree Corners, USA) and with a single-plex ELISA (ELH-HGB, Raybiotech Inc, Peachtree Corners, USA). Un-matched serum and DBSs were assessed using a quantitative multi-plex immunoassay to determine the expression profile of 1000 proteins (QAH-CAA-X00, Raybiotech Inc, Peachtree Corners, USA) and samples collected from the same person were assessed in parallel in a single-plex ELISA (ELH-CA125, ELH-CA724, Raybiotech Inc, Peachtree Corners, USA).

### Statistical methods

Correlation between intra-assay variability results was evaluated using Pearson’s correlation coefficient (r). ANOVA was used to determine the difference between strip blood volume. Matched DBS, serum, and DSS correlation among groups and DBS correlation with hemoglobin were determined with Passing–Bablok regression. Statistical analyses were performed with the R software version 3.5.1 (http://www.R-project.org) and SigmaStat (StataCorp, College Station, USA). In all cases, a p-value < 0.05 was considered as statistically significant.

### Institutional Review Board statement

The study was conducted according to the guidelines of the Declaration of Helsinki and was approved by IRB (ID# 8291-BZhang) and Institutional Human Ethics Committee of the Shunde Hospital of Guangzhou University of Chinese Medicine approved this study (approval no. KY-2020001). Written informed consent was obtained from all subjects involved in the study.

## Results

### Volumetric sampling using shaped filter paper for collection of a defined amount of blood

We describe here a simple and precise approach to the volumetric sampling of DBSs that demonstrates all the advantages of DBS collection while overcoming the sampling issues of hematocrit and homogeneity. Our approach involves the absorption of a liquid capillary blood sample onto a precisely shaped strip of Whatman filter paper, wherein the volume absorbed is controlled by the properties and size of the strip. This approach offers simpler sample collection than traditional DBS card collection and the entire collected sample is analyzed, which allows better integration into sample processing procedures.

**Figure 2:**
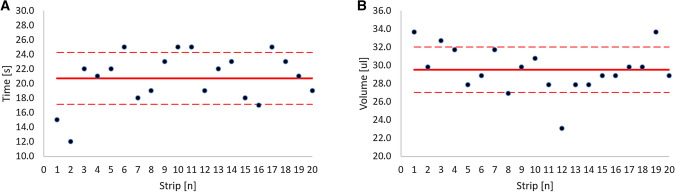
Variation in the fill time and volume of blood absorbed onto the volumetric sampling device. (**A**) complete filling time for 20 strips. (**B**) amount of blood absorbed by 20 strips. Solid red line: represents the average. Dotted red lines: represent the standard deviation.

Several types and sizes of filter paper were analyzed for suitability for the sampling device, including Whatman 903, CF12, and 415, Munktell TFN, and Ahlstrom 226 in 7.5 mm × 7.5 mm and a 5 mm × 10 mm sizes. We found no significant differences between the filter papers tested by size in the filling times and volume (data not shown). The expression of 22 protein biomarkers across 4 strips collected from the same person were assessed in Whatman 903 5 mm × 10 mm strips, Asanté™ 60 mm^2^, and Asanté™ 100 mm^2^ strips (1819-100, 1820-100, Sedia Biosciences, Beaverton, USA). The %CV were 12%, 19% and 16%, respectively (Supplemental Table S6). Thus, the 5 mm × 10 mm size was selected due to the smaller volume requirements compared to the other sizes tested and the Whatman 903 paper was selected due to the low %CV, accessibility, and common use among other CE/IVD available collection devices. The developed volumetric sampling device consists of a 30 mm × 5 mm Whatman 903 filter paper strip secured in a holder that exposes a 10 mm × 5 mm section that collects a 30 µL volume of blood (Fig. 1). The device is dipped into blood samples and absorption occurs by wicking. The strip is defined as full when no visible white areas are observed in the 10mmx5mm exposed area. The filling time for each strip was assessed in 20 strips and found to take 20.70 ± 3.55 s to fill (Fig. 2A). Filling time was dependent on the individual subject and the area of the strip that was immersed in the blood. The strip holder ensures that the tips of each strip do not touch each other or their surroundings during collection and drying, which prevents blood contamination and transfer. The filled strips were allowed to dry for at least 2 h and dried strips were stored at room temperature sealed in a biohazard bag with a desiccant pack.

The exact volume of blood absorbed by the device was assessed by weighing the blood absorbed in 20 strips. Each strip was found to absorb 29.52 ± 2.48 µL of blood (Fig. 2B). The coefficient of variation in volume of blood absorbed was 8.39%, which is much less than the 30% variation in the volume of blood collected using a traditional DBS card and punch approach^39^ and is comparable to the 5% and 8.1% variation found in similar volumetric sampling devices^31,32^. We then assessed whether it was possible for the device to be overfilled. Ten strips were allowed to absorb blood until visibly saturated, then the strips were exposed to 0, 1, 2, 3, or 4 additional drops of blood. The volume of blood absorbed by the device was then assessed by weighing as described above. There was no significant difference in the volume of blood absorbed in strips that were exposed to additional drops of blood compared with strips not exposed to additional blood (p = 0.22), indicating that overfilling the strip did not occur in this situation (data not shown).

One of the advantages of DBSs is the ability to obtain high quality samples from remote locations and/or developing countries and from those with limited access to clinics, such as military patients or patients with limited mobility such as the elderly. Our volumetric sampling device allows for remote, in-home blood collection and sample shipment by mail, which facilitates DBS expansion into these populations. To investigate the accuracy of remote collection from naïve users, we assessed operator variability in the volume of blood sampled. The accuracy of blood volume collected and ease-of-use of our device was assessed in samples collected by 16 operators with no experience in the collection of blood samples and who had not previously used this or any DBS collection device. The operators were given written instructions for the use and collection of a DBS with the device. Each operator attempted to fill 5 strips. Of the 80 samples collected, 63 (78.75%) were properly filled with blood according to the instructions. These results are in good agreement with the sampling errors associated with other established techniques of DBS collection using naïve collectors^40^. Operator feedback indicated that the instructions were relatively clear and easy to follow.

To verify the volumetric sample device developed here overcame the hematocrit issues seen in traditional DBS cards, we analyzed the expression of 200 proteins as well as hemoglobin concentration in 21 matched serum, DBS, and dried serum samples (DSS) samples. The hemoglobin concentration was 32.00 ± 1.57 µg/mL, 148.70 ± 11.41 mg/mL, and 24.30 ± 1.30 µg/mL in serum, DBS, and DSSs, respectively, which is consistent with published levels^41^. The average correlation of the expression of 200 proteins across DBS/DSS, DBS/Serum, DSS/Serum, Hemoglobin/DBS, Hemoglobin/DSS, and Hemoglobin/Serum groups in matched serum, BDS, and DSS sample with hemoglobin concentration were 0.63, 0.56, 0.67, − 0.08, − 0.07, and − 0.10, respectively. These data show that the correlation of hemoglobin concentration with the expression of each of the 200 proteins assessed in DBS, DSS, and serum was negligible. Indicating that hematocrit as evidenced by the hemoglobin concentration is not an issue with determining the quantitative protein expression using our volumetric sampling device.

We have demonstrated that this volumetric sampling device is capable of collecting an accurate, reproducible volume of blood that can be used for quantitative analysis, independently of hematocrit and homogeneity issues. This device can also be used to provide quantitative analysis from samples collected from populations not normally included in clinical study. While this device addresses some of the major disadvantages of DBSs for clinical quantitative analysis, a thorough comparison of the DBS method against venous blood draw is needed. Preferably, this comparison would be conducted across a wide range of protein analytes before DBSs may be deemed comparable or not to liquid serum and/or plasma.

### Validation and optimization of volumetric DBS device procedure for quantitative proteomics analysis

A thorough method validation requires a strict protocol covering all relevant assay steps. Thus, a comprehensive workflow for preparing DBSs collected with our volumetric sampling device and analysis with a quantitative proteomic immunoassay for future use in a clinical setting is presented, including (1) the collection of blood, (2) drying and storage, (3) elution of DBSs from the strips, (4) optimization of the procedure, and (5) analyses of DBS eluates.

**Figure 3:**
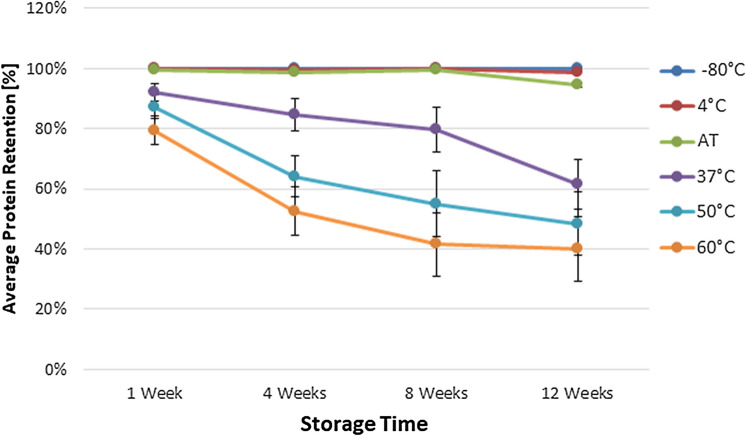
Average stability of 14 proteins in DBS samples over 3 months at varying temperatures. The average retention in the expression levels of 14 relatively serum abundant proteins in DBS samples stored at − 80 °C, 4 °C, ambient temperature (AT), 37 °C, 50 °C, and 60 °C for 1 week, 4 weeks, 8 weeks, and 12 weeks were compared with the expression levels from a corresponding freshly collected DBS sample. Data are expressed as the mean ± SEM.

One of the advantages of DBSs is their stability in storage, enabling the shipment of the dried samples at ambient temperature. We investigated the stability of 14 relatively abundant serum proteins (listed in Supplemental Table S1) in DBSs using an accelerated stability test protocol. A total of 25 DBSs were collected with our volumetric sampling device and stored at − 80 °C, 4 °C, ambient temperature (AT), 37 °C, 50 °C, and 60 °C for 0, 1, 4, 8, and 12 weeks. The expression levels of the 14 analytes were quantitatively assessed and compared with the expression levels from a corresponding freshly collected DBS (Fig. 3). Average protein retention over 12 weeks was 100%, 99%, 95%, 62%, 48%, and 40% for DBSs stored at − 80 °C, 4 °C, AT, 37 °C, 50 °C, and 60 °C, respectively. Our results show that a small volume of blood (30 µL) can be used to quantitatively assess 14 proteins and these samples can be stored at ambient temperature for up to 3 months with no significant loss of protein stability.

**Figure 4:**
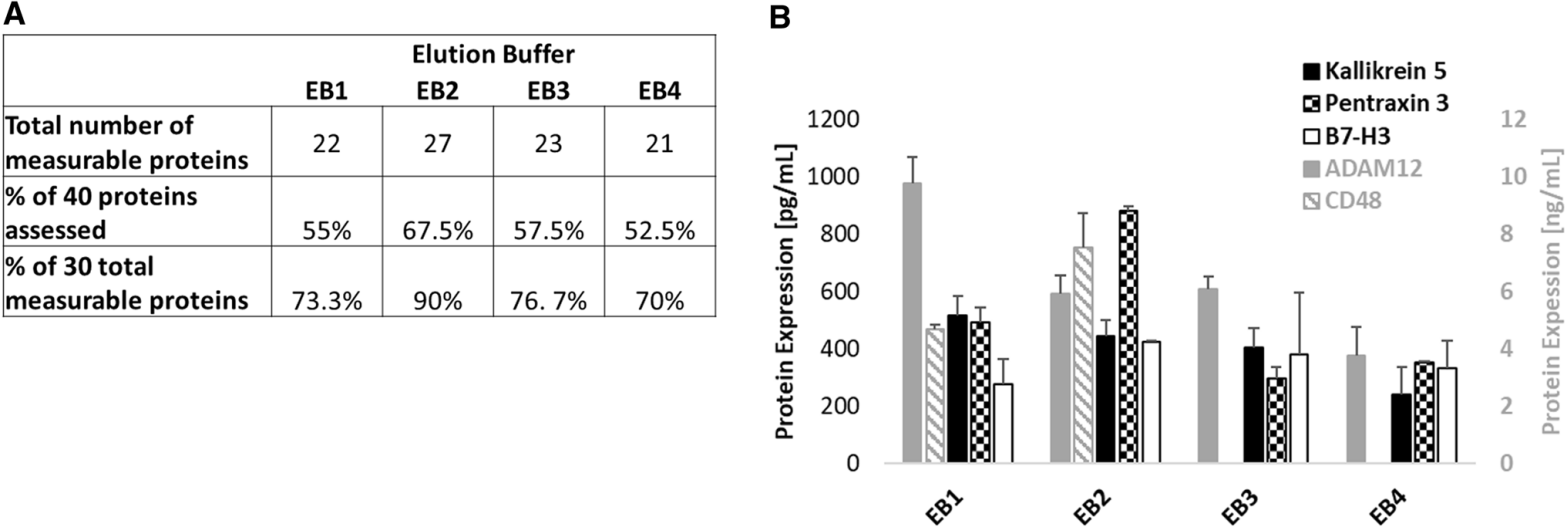
Optimization of Elution buffer composition for protein extraction in DBS samples. 4 different elution buffers: 1 × PBS (EB1), 1 × PBS containing 0.1% Tween-20 (EB2), 1 × PBS containing 10% casein and 50% BSA (EB3), and 1 × PBS containing 10% casein, 50% BSA, and 0.1% Tween-20 (EB4) were used to elute 3 DBS samples each. (**A**) The number and percentage of 40 proteins that were quantitatively assessed and measurable with each elution buffer. (**B**) Concentrations and variance as evidenced by the standard error of 5 proteins compared across the 4 different elution buffers.

Once the blood is collected and stored, the next step in the method is elution of the DBS from the sampling device. For many clinical applications, accurate measurement of multiple proteins is needed. The limitation of a small sample volume requires efficient use of all DBS material collected. Similarly, the extraction efficiency of the proteins from the densely dried sample is paramount. Various proteins and molecules diffuse at different rates, more or less efficiently, from the collection device and may cause a variable and thus less accurate recovery rate^42–44^. Thus, optimization of the elution method used is necessary for the end assay application. To optimize and validate the elution of proteins from the entire DBS collected with our volumetric sampling device, we analyzed 4 different elution buffers: 1 × PBS (EB1), 1 × PBS containing 0.1% Tween-20 (EB2), 1 × PBS containing 10% casein and 50% BSA (EB3), and 1 × PBS containing 10% casein, 50% BSA, and 0.1% Tween-20 (EB4). The completely filled 5 mm × 10 mm exposed section of the strip was cut using straight, clean surgical scissors and placed into a 1.5 mL conical tube using forceps cleaned with alcohol. The elution buffer was added to the conical tube at a 1:10 ratio and eluted for 4 h at room temperature on a shaker, vortexing for 10 s every 30 min. Next, the liquid was transferred to a clean 1.5 mL conical tube leaving the strip behind and centrifuged at 14,000 rpm for 10 min. The supernatant was collected into a clean 1.5 mL conical tube. We then quantitatively assessed 40 proteins (listed in Supplemental Table S2) in 3 DBSs each. These 40 proteins were chosen partially based on the wide variation in expression levels in serum reported in the literature. Ten of the 40 total proteins assessed were not detectable using any elution buffer in these particular samples. The 30 remaining proteins were detectable in at least 1 elution buffer. Of the remaining 30 proteins, 22, 27, 23, and 21 were detectable using EB1, EB2, EB3, and EB4, respectively (Fig. 4A). The concentrations of 5 of those proteins, ADAM12, B7-H3, CD48, Kallikrein 5 and Pentraxin 3 with elution using all 4 buffers are also shown (Fig. 4B). Both the concentrations and variation (shown as standard error across samples) were different for each protein with each elution buffer. Overall, for the 30 measurable proteins, EB2 had the least variance in protein concentration across samples tested compared to the other elution buffers. Our results also indicated that EB2 allowed for the quantitative measurement of the most proteins. Thus, this elution buffer was used in all following assays.

**Figure 5:**
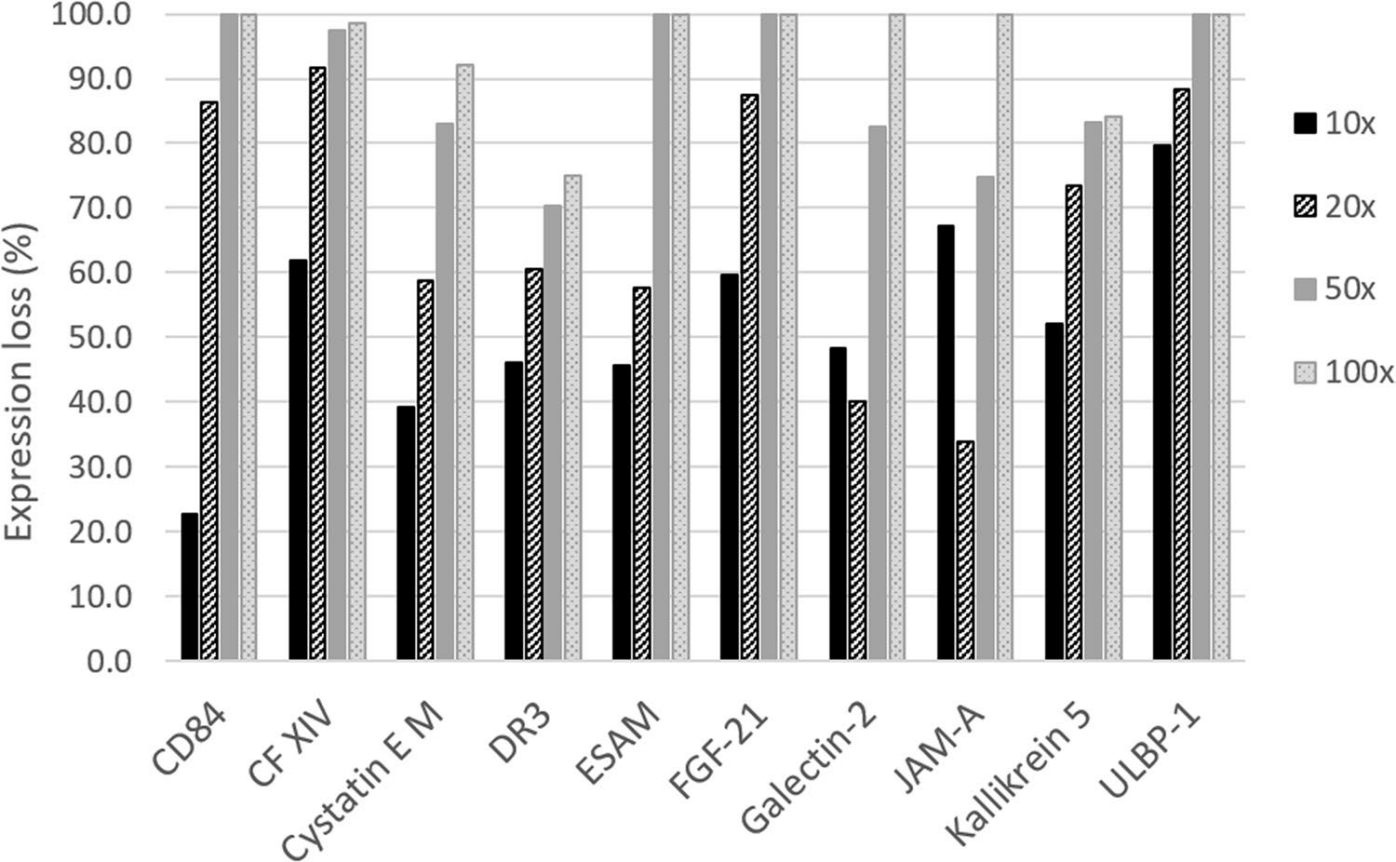
Optimization of sample dilution of DBS eluates for use on a quantitative immunoassay platform. 5 different eluate dilutions: 5 ×, 10 ×, 20 ×, 50 × and 100 × were used to quantitatively assess 40 proteins. The % expression loss compared with the 5 × dilution is shown for a subset of 10 proteins.

We further optimized the sample dilution of the DBS eluates for use on the quantitative immunoassay platform using the provided assay sample diluent. A total of 15 DBSs were collected and eluted using EB2. Five different dilutions of the eluate were tested in triplicate: 5 ×, 10 ×, 20 ×, 50 × and 100 ×, with quantitative assessment of the same 40 proteins as above. Results show that the total number of measurable proteins was 37, 33, 29, 25, and 21 for the 5 ×, 10 ×, 20 ×, 50 × and 100 × dilutions, respectively. In choosing the optimal dilution, we took into consideration the following: background signals across the multi-plex array (which were increased in the 5 × dilution group, data not shown), the amount of protein expression retained as the eluate dilution increased (Fig. 5), and the total sample volume restriction, which would make quantification of a large number of proteins untenable at low eluate dilutions. We concluded that a 20 × eluate dilution yielded the best signal to background ratio. Thus, a 20 × eluate dilution in EB2 was used for all further assays.

**Figure 6:**
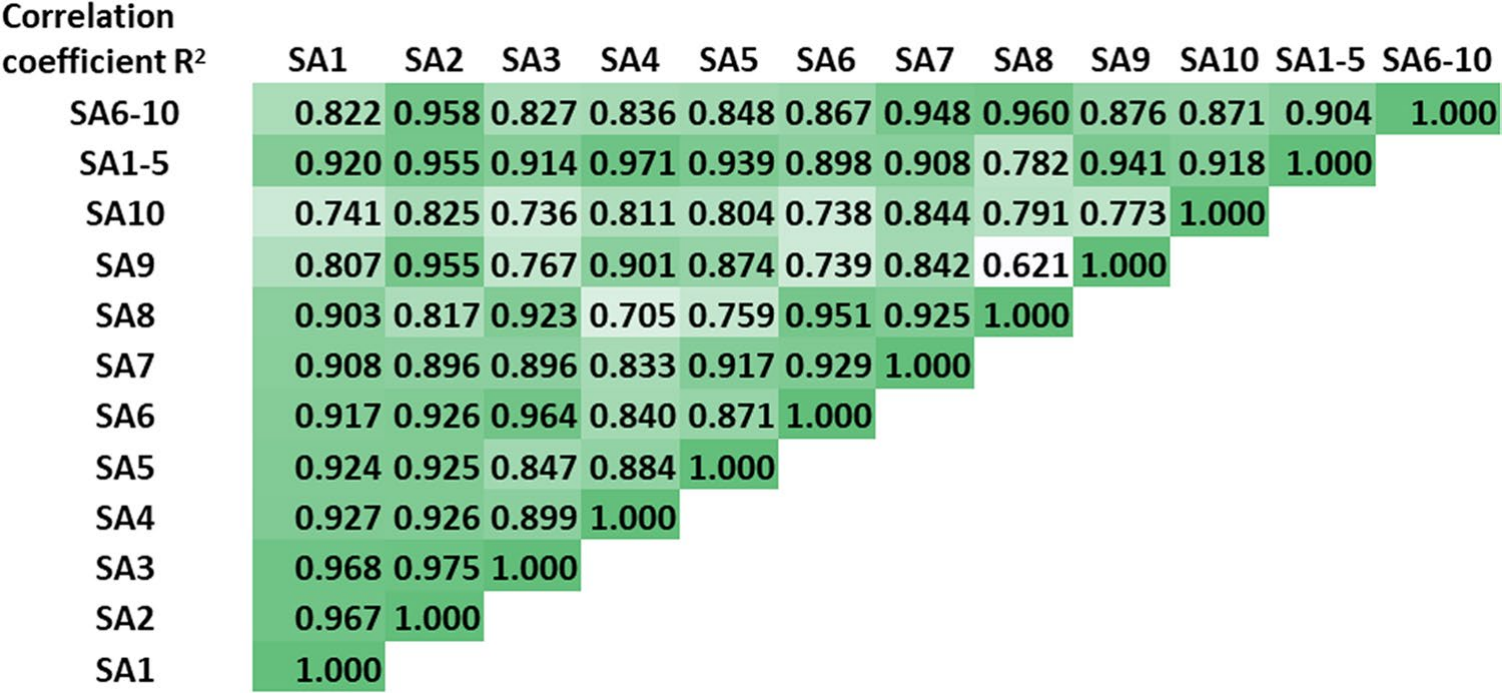
Accuracy and reproducibility of the quantitative proteomics analyses A. correlated protein expression across sample (SA) replicates 1 through 10 and pooled samples (SA1-5 and SA6-10).

To investigate the accuracy and reproducibility of the quantitative proteomics analyses (intra-assay variability) we collected a DBS using our volumetric sampling device from a single individual at a single time-point and preformed the entire validated method indicated above 10 separate times. We correlated protein expression across the replicates and found the mean r^2^ correlation value between replicates was 0.860 with a range of 0.621–0.975 (Fig. 6). Average within-subject % CV across the quantitative assessment of the measurable proteins (29 out of 40) was 23.77% across 10 tests (Table 1). We also assessed variability across individual samples based on collection order. Ten individual DBSs were collected from a single individual and numbered based on the order they were collected. A pooled sample of the first 5 DBSs collected and of the last 5 DBSs collected were also assessed. The levels of protein expression for each sample were compared to each other and to the pooled samples. We found no difference in protein expression based on collection order or between independent and pooled samples (Fig. 6). These results indicate that collection order does not compromise reproducibility, and that any number of samples can be collected and pooled to generate enough sample volume to test any number of proteins, with subject comfort and physical bleeding capacity being the only limiting factors.

**Table 1:** Intra-assay variability. Average within-subject % CV from 10 independent DBS strips. Eluate from each strip was run on a multi-plex array capable of measuring 40 proteins. 29 out of the 40 proteins were measurable, and the % CV is presented for each protein. The overall average % CV across all 29 proteins was also presented. ND: not detectable.

Protein name	% CV	Protein name	% CV	Protein name	% CV
ADAM8	8.80	Cystatin A	42.21	Midkine	25.08
ADAM12	14.89	Cystatin B	19.36	Pentraxin 3	28.22
B7-H3	12.75	Cystatin E M	10.82	Pref-1	14.05
BMPR-IB	33.80	Desmoglein 2	22.31	Siglec-10	23.36
Cadherin-4	ND	DR3	38.63	SLAM	ND
Cadherin-13	28.45	ErbB4	ND	SP-D	18.82
CD48	22.45	ESAM	ND	Syndecan-4	15.89
CD58	50.11	FGF-21	18.56	Testican 2	ND
CD84	44.74	Galectin-2	12.58	TIM-3	ND
CD99	16.83	Galectin-9	7.20	TLR4	ND
CD155	29.51	ICOS	26.26	TRAIL	ND
CD229	ND	JAM-A	30.26	ULBP-1	ND
CEACAM-5	26.03	JAM-B	ND	Average %CV	23.77
CF XIV	17.29	Kallikrein 5	30.15		

### Analysis of 1000 protein analytes in DBSs with an established quantitative multi-plex immunoassay

To establish confidence in and implementation of DBSs in clinical applications, validation of a large number of proteins that span all levels of expression, using an approach that has been previously clinically established, is necessary. Although we are not validating the quantitative multi-plex used here clinically, the potential to do so for a subset of identified proteins of interest is available in the future.

**Figure 7:**
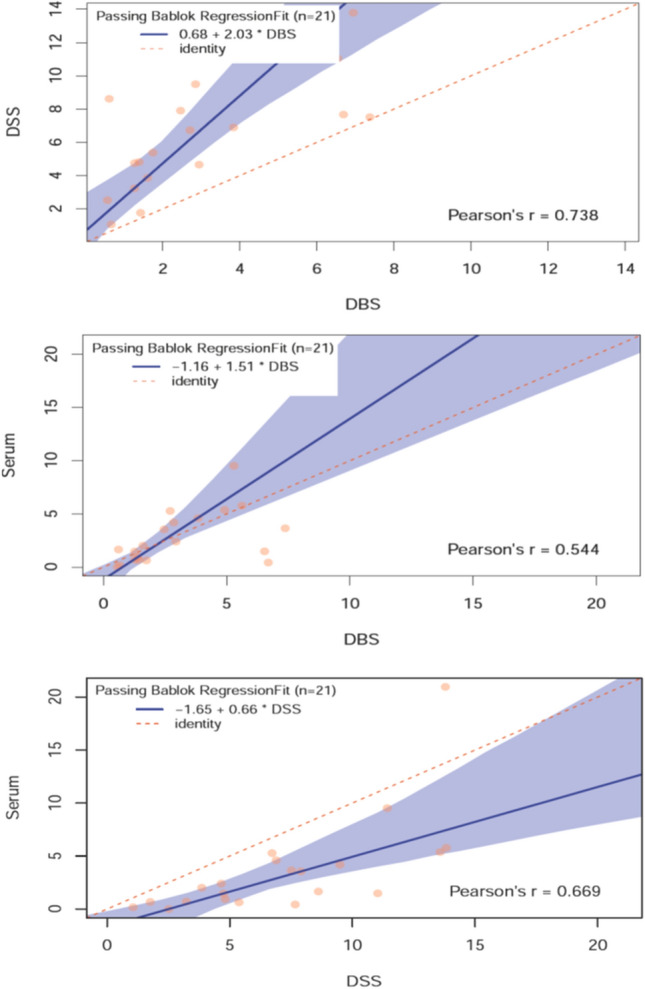
Example Passing–Bablock regression of the expression of 200 proteins across groups in matched serum, DBS, and DSS samples for MCP-4.

First, we tested 21 matched serum, DBS, and DSSs using quantitative multi-plex immunoassay to determine the expression profile of 200 proteins. Of these 200 proteins, 18 were undetectable (i.e., below the limit of detection (LOD) in at least 2/3 of all samples per group) in all sample types (G-CSF, GM-CSF, I-309, IFNγ, IL-1α, IL-2, IL-4, IL-6, IL-7, IL-8, IL-10, IL-11, IL-12p70, IL-17, TNFβ, BMP-4, GRO, and IL-2 Rγ). Another 9 were undetectable in DBS (IL-5, IL-13, MCSF, TNFα, bFGF, BMP-7, FGF-7, IGF-1, and XEDAR), 6 were undetectable in DSS (IGF-1, XEDAR, IL-1β, GITR, IL-21R, and NRG1-β1), and 18 were undetectable in serum (IL-5, IL-13, MCSF, TNFα, IL-1β, NRG1-β1, IL-1ra, MCP-1, MIP-1β, TGFβ3, VEGF, CTACK, IL-9, IL-29, I-TAC, LIF, CD30, and Contactin-2). As shown in Fig. 7, there is a moderate correlation among the proteins assessed across the three sample types. DSS/serum had the highest correlation, with 69% of the measurable proteins having a Pearson’s r value greater than 0.5. The percentage of measurable proteins having a Pearson’s r value above 0.5 for DBS/DSS and DBS/serum are 60% and 55%, respectively. However, among all matched sample group comparisons, the correlations ranged wildly, with a number of individual proteins that were not correlated (Pearson’s r value < 0.3) and a number of individual proteins that were highly correlated (Pearson’s r value > 0.7). The concentrations and Passing–Bablok regressions for each of the measurable proteins is shown in Supplemental Table S4 and Supplemental Table S5, respectively. These results indicate that a sweeping comparison of sample types for expression of all proteins is likely to be inaccurate and that each sample type should be assessed independently for clinical suitability, or a larger validation of each individual sample type on its correlation with another should be conducted for each independent protein.

**Figure 8:**
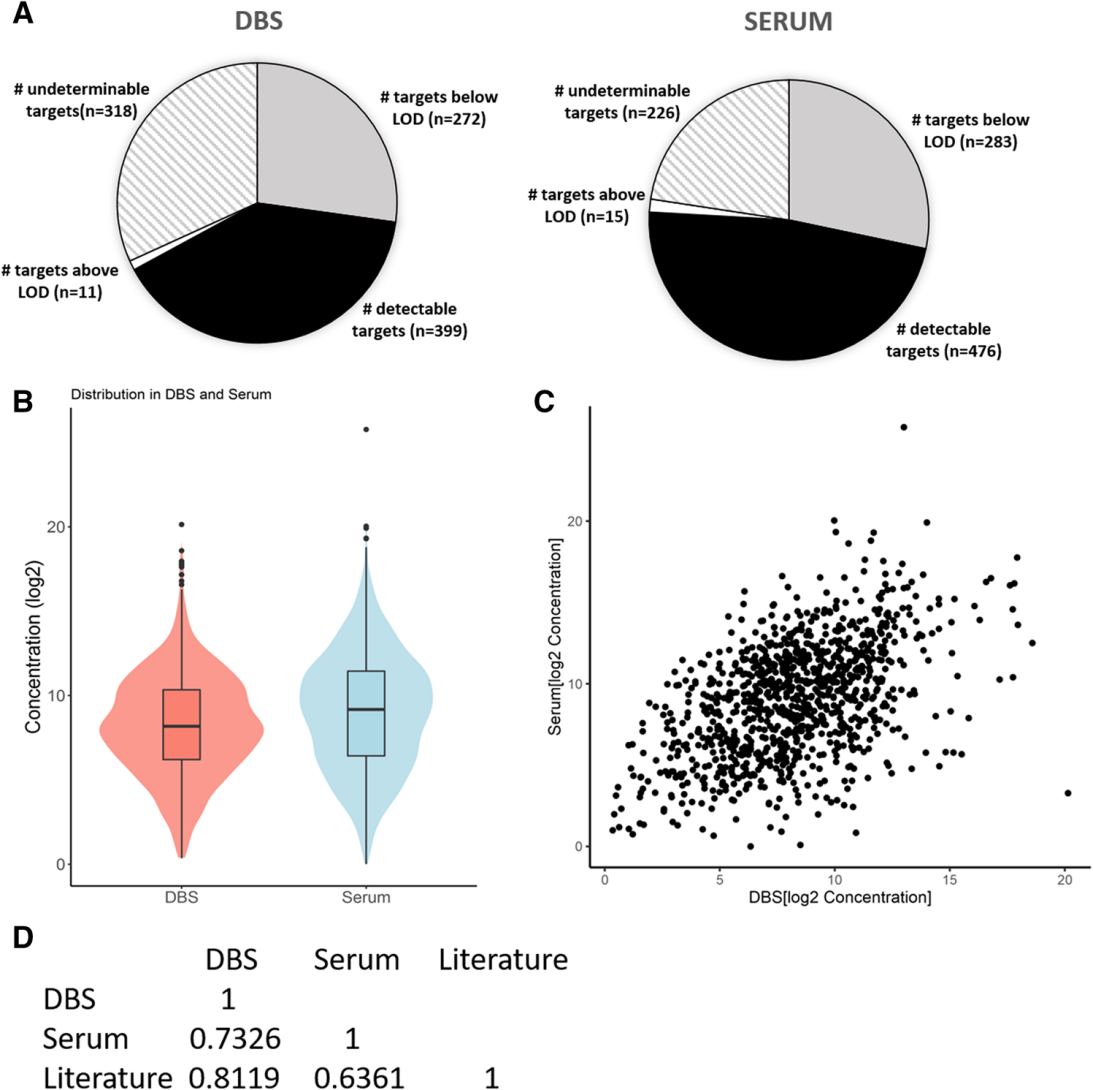
Assessment of measurable proteins in normal DBS and serum samples in a quantitative multi-plex immunoassay. (**A**) Summary of the number of 1000 proteins that are below the LOD, within the detectable range, above the LOD, or that didn’t fall completely in either of the aforementioned categories in at least 2/3 of all samples assessed in DBS and serum, respectively. (**B**) Distribution of the concentration of measurable proteins in DBS and serum samples by violin plot. (**C**) Scatterplot of the distribution of the concentration of measurable proteins in DBS and serum samples. (**D**) Correlation of the 18 DBS and 28 Serum measurable FDA-approved protein biomarkers in at least 2/3 of the 72 DBS and 200 serum samples assessed with each other and reported serum literature values.

Next, we tested unmatched serum and DBSs in parallel using a quantitative multi-plex immunoassay to determine the expression profile of 1000 proteins^45^. The list of the 1000 proteins analyzed can be found in Supplemental Table S3 and the validation linearity and recovery data for the assay can be found in the manufacturer’s protocol. Two hundred normal serum samples and 72 normal DBSs were collected. Of the 1000 proteins analyzed, 410 were measurable (i.e., above the limit of detection (LOD)) in at least 2/3 of all the DBSs assayed, whereas 491 were measurable in at least 2/3 of all the serum samples assayed (Fig. 8A). A similar number of targets, 283 and 272 targets in DBSs and serum samples, respectively, were below the LOD in at least 2/3 of all samples analyzed. 318 and 226 targets in DBSs and serum samples, respectively, did not clearly fall in either condition (measurable or below the LOD) in at least 2/3 of all samples, making it difficult to clearly define the measurability of those targets. The distribution of concentrations of all the measurable proteins in both serum and DBSs is shown in Fig. 8B,C. To date, there are over 100 protein biomarkers approved by the US FDA for clinical use in serum/plasma^46^. In total, 40 of the FDA-approved protein biomarkers were present in the tested 1000 protein dataset. We tested whether these proteins are also measurable in DBSs. Of the 40 FDA-approved markers tested, 18 were measurable in at least 2/3 of all DBSs tested, whereas 28 were measurable in at least 2/3 of all serum samples tested. Expression levels for these 18 and 28 targets, respectively, were reasonably well correlated with normal serum values reported in the literature despite the differences in sample type (in the instance of DBSs), individuals tested, and quantification methods (R^2^ value of 0.81 and 0.64 for DBSs and serum samples, respectively) (Fig. 8D). Validation in single-plex assays for clinical use is often required for biomarkers identified in large scale, multi-plex analyses. Similarly, there are a number of well-known biomarkers, including cancer markers CA72-4 and CA125, that are often used independently in single-plex assays. Therefore, we tested 5 matched DBS and serum samples on single-plex ELISAs for CA72-4 and CA125 (Table 2). The results showed good correlation between serum and DBS for these 2 proteins (adjusted R-squared = 0.7002 and 0.9717 for CA125 and CA72-4, respectively) suggesting that for some markers, DBS may be capable of being used as an alternative sample type for clinical analyses for those patients in which serum collection is contraindicated using the serum values. However, many of the 1000 proteins tested were found to not correlate well between sample types, indicating that DBS should be treated independently from serum when assessing clinical suitability. Similarly, when determining if the clinical serum values are suitable for a DBS, each protein should be validated for correlation independently.

**Table 2:** Protein expression and correlation of protein expression of 5 matched serum and DBS samples run on single-plex ELISAs for CA125 and CA72-4.

	CA125 (U/mL)		CA72-4 (U/mL)	
	DBS	Serum	DBS	Serum
Sample 1	1.42	5.62	2.04	1.59
Sample 2	1.42	7.97	2.04	3.34
Sample 3	2.06	7.27	2.50	8.91
Sample 4	3.78	15.99	1.83	1.76
Sample 5	2.13	5.26	3.20	19.09
	*Adjusted R-squared* = *0.7002*		*Adjusted R-squared* = *0.9717*	
	*p-value* = *0.0487*		*p-value* = *0.0013*	

## Discussion

We show here that our volumetric sampling device is capable of collecting an accurate, reproducible volume of blood that can be used for quantitative analysis of DBSs. The quantitative assessment of 1000 proteins with a multi-plex immunoassay platform also provides a first step in the establishment of a DBS database for the broad application of this sample type in clinical applications. Further studies comparing different clinical populations and target proteins will determine if this is a valid approach for use in all clinical applications.

The use of DBSs has many advantages, including ease of use (minimally invasive sample collection, small sample volume, minimal sample processing requirements, and ease of automation), low cost, long-term stability in shipping or storage, and increased feasibility for remote sampling. Yet the impediments of small sample volume, area bias, and homogeneity issues have limited the clinical utility of DBSs. The small volume decreases sensitivity and accuracy of quantitation, while non-uniform blood distribution across a DBS and changes in hematocrit levels can arise due to collection volume uncertainty. Non-homogeneity across a DBS sample have been addressed with a variety of approaches, including whole spot analysis and volumetric sampling^30–37,47,48^. We have developed a specially designed volumetric blood collection device which yields a sufficient quantity of sample to test any number of proteins in a multi-plex immunoassay, with bleeding capacity being the most significant limiting factor of sample volume. Additionally, our volumetric sampling device utilizes the entire blood sample, allowing for better quantitation of data and more proteins to be assessed. Previous studies have demonstrated that the hematocrit issues found with traditional DBS cards is effectively eliminated with a volumetric sampling device^49^. In this study, we also found hematocrit, as evidenced by the hemoglobin concentration, is not an issue with determining the quantitative protein expression using our volumetric sampling device. This method also allows a controlled volume of blood to be collected, which minimizes area bias and heterogeneity. Although volumetric sampling devices like the one developed here overcame certain limitations of traditional DBS collection, to date there is no consensus about the preparation and processing of DBS samples, which is necessary to transform DBS to a practical clinical strategy for proteomic and other analyses.

We considered several steps in the sample processing protocol, including design of the elution buffer and sample dilution. A number of studies have used PBS either with or without a stabilizing protein (e.g. BSA or Tween-20)^13,50^. In this study, we directly compared elution buffers with various combinations of PBS, casein, BSA and Tween-20. We observed relatively similar elution capabilities for all buffers but determined PBS + 0.1% Tween-20 to be optimal for the multi-plex immunoassay being tested. We also tested five different sample dilutions. While a 5 × dilution was optimal when considering measurability of the highest number of proteins, additional factors such as non-specific reactivity, dilution sensitivity, and total volume restrictions vs. total number of proteins assessed led us to conclude that a 20 × dilution was optimal for our immunoassay. Optimization of the elution conditions for each target protein was not possible due to the parallel nature of the multi-plex assay we employed as well as the limited sample volume available. Consequently, measurability of some target proteins may have been sacrificed in the interest of high-content data. In future studies, the particular application being investigated should be carefully considered when selecting DBS processing procedures.

Multi-plex enzyme immunoassays are well-established methods for quantifying large numbers of proteins in a small amount of sample with high sensitivity and specificity^51,52^. The low costs and commercial availability of these assays also make them appealing for further expansion into clinical applications^53,54^. A quantitative immuno-assay was assessed here since such an approach has been previously clinically validated and would be suitable for such in the future. However, this application does have limitations in reproducibility and non-linearity, especially close to the LOD. Using a quantitative multi-plex immunoassay approach, we showed that 410 out of 1000 proteins were measurable in at least 2/3 of all 72 DBSs assayed, including 18 FDA-approved protein biomarkers. We further showed that in at least 2/3 of the 200 serum samples tested, 491 proteins were measurable, including 28 FDA-approved protein biomarkers. While the intra-assay variability had a mean r^2^ correlation value of 0.860, the average within-subject % CV was 23.77% across 10 tests. These 10 tests exhibited significant subject-to-subject variability, such that 318 and 226 targets in DBSs and serum samples, respectively, were not either measurable or below the LOD in at least 2/3 of all samples, making it difficult to clearly define the measurability of those targets. To measure biological variations in the levels of protein biomarkers within an individual over time, the variation in the assay should be ideally < 0.5^55^,which falls in the range of 2–10% total assay CV for most proteins^56^. According to the manufacturer, the assay specific CV for the multiplex immunoassay used is already 10–15%. The sample processing procedure as well as specific protein characteristics, e.g., stability, size, structure, etc. can add further variation to the analyses. Thus, further optimization is required to reduce the variability both within and across subjects to improve the application of this approach for more target proteins and for clinical use.

In this study, we optimized the DBS method concomitantly with serum with a common immunoassay-based, quantitative proteomics approach for 1000 proteins. We noted several detection differences between serum and DBS: 1. despite the ability to utilize the entire sample collected with our device, the small DBS volume restricted the total number of detectable proteins, 2. some low-abundance proteins cannot be detected in either sample type with this assay, and 3. many differences in protein measurability across sample type did not correlate with relative expression level. For example, the low-abundance protein ULBP-1 was detected at comparable levels in DBS (41 pg/mL) and serum (90 pg/mL), whereas more abundantly expressed proteins were not detectable in one or the other sample type (e.g., ErbB4 which had serum levels of 7.8 ng/mL compared with undetectable levels in DBS; VEGF-A which had serum levels that were undetectable whereas DBS levels were 225 pg/mL). Additionally, some analytes were detected in both sample types but at significantly different levels (e.g., ACE serum levels equal to 48.2 ng/mL compared with DBS levels equal to 9.1 ng/mL). While this comparison helped optimize the DBS protocol and demonstrated the validity of DBS as a suitable clinical sample type for proteomic analyses, it suggests that serum and dried blood are distinctly different sample matrices, with direct comparisons between them unlikely to be beneficial. Despite some good correlation being noted for some well-known protein biomarkers (i.e., CA125 and CA72-4), clear differences between serum and plasma matrices for several protein biomarkers are well known, so it is not unexpected that differences exist for serum and dried blood. Thus, a separate validated protein expression database for DBS independent of serum or plasma, where normal and disease levels are known, is required before DBS will be fully implemented in a wide range of clinical applications.

This study is one of the first attempts to build a proteomic dataset for DBSs necessary to establish successful, widespread clinical implementation. Further studies aimed at reducing the % CV observed within and across subjects are needed, as well as further optimization of the protocol to maximize the number of detectable proteins spanning multiple levels of expression. Finally, expanding the underdeveloped DBS database for clinical applications should be conducted. It is our expectation that further application of quantitative proteomic techniques will facilitate these studies and expedite the validation of DBSs as a clinically relevant sample type.

## Supplementary Information

Supplementary Information.

## Data Availability

Please address all data requests to the corresponding author.

## Abbreviations

DBS: Dried blood sample
ELISA: Enzyme-linked immunosorbent assay
PVC: Polyvinyl chloride
AT: Ambient temperature
LOD: Limit of detection
IVD: In vitro diagnostic
DSS: Dried serum sample
