# Supplementary material for: Dried blood sample analysis by antibody array across the total testing process
Source: Sci Rep. 2021 Oct 15;11:20549. doi: 10.1038/s41598-021-99911-8 (PMC8521592; doi:10.1038/s41598-021-99911-8)
Supplement: Supplementary file 1 — Supplementary Information. [file 41598_2021_99911_MOESM1_ESM.pdf]

Supplemental Data

Angiotensinogen
CHI3L1
Dkk-3
ICAM-1
IL-6R
LAP(TGFb1)
NAP-2
PARC
P-Cadherin
PGRP-s
RANTES
Siglec-5
TIMP-1
TIMP-2

Table S1: the 14 proteins used for stability assessment

ADAM8	ESAM
ADAM12	FGF-21
B7-H3	Galectin-2
BMPR-IB	Galectin-9
Cadherin-4	ICOS
Cadherin-13	JAM-A
CD48	JAM-B
CD58	Kallikrein 5
CD84	Midkine
CD99	Pentraxin 3
CD155	Pref-1
CD229	Siglec-10
CEACAM-5	SLAM
CF XIV	SP-D
Cystatin A	Syndecan-4
Cystatin B	Testican 2
Cystatin E M	TIM-3
Desmoglein 2	TLR4
DR3	TRAIL
ErbB4	ULBP-1

Table S2: the 40 proteins assessed in the determination of the optimal elution buffer and sample dilution

Table S3: the 1000 proteins assessed in the parallel comparison of 200 serum and 72 DBSs

Target	Accession Number
17-beta-HSD1	P14061
2B4	Q9BZW8
4-1BB	Q07011
4-1BB Ligand	P41273
5'-Nucleotidase	P21589
6Ckine	O00585
ACE	P12821
ACE-2	Q9BYF1
Activin A	P08476
Activin R1A	Q04771
Activin R1B	P36896
Activin R2A	P27037
Activin R2B	Q13705
ADA	P00813
ADAM-12	O43184
ADAM-22	Q9P0K1
ADAM-23	O75077
ADAM-8	P78325
ADAM-9	Q13443
ADAMTS-13	Q76LX8
ADAMTSL-1	Q8N6G6
Adiponectin	Q15848
Adipsin	P00746
ADP-Ribosyl Cyclase 2	Q10588
aFGF	P05230
Aggrecan	P16112
AgRP	O00253
AIF	O95831
AKR1C4	P17516
Albumin	P02768
ALCAM	Q13740
ALK-1	P37023
ALK-6	O00238
Alpha-fetoprotein	P02771
AMICA	Q86YT9
AmiGO	Q86WK6
AmiGO2	Q86SJ2
Aminopeptidase LRAP	Q6P179
Aminopeptidase P2	O43895
Amnionless	Q9BXJ7
Amphiregulin	P15514
AMSH	O95630
Angiogenin	P03950
Angiopoietin-1	Q15389
Angiopoietin-2	O15123
Angiopoietin-4	Q9Y264

Angiostatin	P00747
Angiotensinogen	P01019
ANGPTL3	Q9Y5C1
ANGPTL4	Q9BY76
ANGPTL7	O43827
Annexin A5	P08758
APRIL	O75888
Arginase 1	P05089
ARNT	P27540
Artemin	Q5T4W7
Arylsulfatase A	P15289
Arylsulfatase B	P15848
ASAH2	Q9NR71
ASAHL	Q02083
AXL	P30530
B3GNT2	Q9NY97
B4GalT1	P15291
B7-H1	Q9NZQ7
B7-H2	O75144
B7-H3	Q5ZPR3
B7-H4	Q7Z7D3
BAFF	Q9Y275
BAFF R	Q96RJ3
BAI1	O14514
BAMBI	Q13145
BATF3	Q9NR55
BCAM	P50895
BCL-10	O95999
BCL-2	P10415
bcl-w	Q92843
BCMA	Q02223
BDNF	P23560
beta IG-H3	Q15582
Beta-2 Microglobulin	P61769
Betacellulin	P35070
Beta-glucuronidase	P08236
beta-NGF	P01138
bFGF	P09038
BID	P55957
Biglycan	P21810
BLAME	Q9P0V8
BLC	O43927
BMP-2	P12643
BMP-4	P12644
BMP-5	P22003
BMP-7	P18075
BMP-8	Q7Z5Y6

BMP-9	Q9UK05
BMPR-IA	P36894
BMPR-II	Q13873
BOC	Q9BWV1
Bora	Q6PGQ7
Brevican	Q96GW7
Brorin	Q2TAL6
C1qTNF4	Q9BXJ3
C1qTNF9	P0C862
C5a	P01031
CA12	O43570
CA125	Q8WXI7
CA13	Q8N1Q1
CA14	Q9ULX7
CA15-3	P15941
CA19-9	
CA2	P00918
CA4	P22748
CA5A	P35218
CA5B	Q9Y2D0
CA6	P23280
CA72-4	
CA8	P35219
CA9	Q16790
Cadherin-11	P55287
Cadherin-13	P55290
Cadherin-17	Q12864
Cadherin-4	P55283
Cadherin-6	P55285
Calcitonin	P01258
Calreticulin	P27797
Calreticulin-2	Q96L12
Calsyntenin-1	O94985
CANT1	Q8WVQ1
Carboxylesterase 1	P23141
Carboxylesterase 2	O00748
Carboxypeptidase A2	P48052
Carboxypeptidase B1	P15086
Carboxypeptidase E	P16870
Cardiotrophin-1	Q16619
Caspase-7	P55210
Caspase-8	Q14790
Caspr2	Q9UHC6
Cathepsin B	P07858
Cathepsin E	P14091
Cathepsin H	P09668
Cathepsin L	P07711

Cathepsin S	P25774
Cathepsin V	O60911
Caveolin-2	P51636
CCL28	Q9NRJ3
CD109	Q6YHK3
CD14	P08571
CD147	P35613
CD155	P15151
CD163	Q86VB7
CD2	P06729
CD200	P41217
CD200 R1	Q8TD46
CD229	Q9HBG7
CD23	P06734
CD26	P27487
CD27	P26842
CD27 Ligand	P32970
CD28	P10747
CD30	P28908
CD300a	Q9UGN4
CD300c	Q08708
CD300e	Q496F6
CD300f	Q8TDQ1
CD314	P26718
CD320	Q9NPF0
CD34	P28906
CD36	P16671
CD39L2	O75354
CD39L3	O75355
CD39L4	O75356
CD4	P01730
CD40	P25942
CD40 Ligand	P29965
CD42b	P07359
CD48	P09326
CD5	P06127
CD51	P06756
CD58	P19256
CD6	P30203
CD69	Q07108
CD7	P09564
CD80	P33681
CD83	Q01151
CD84	Q9UIB8
CD86	P42081
CD97	P48960
CD99	P14209

CD99-L2	Q8TCZ2
CDC25B	P30305
CDNF	Q49AH0
CDO	Q16878
CEA	P06731
CEA variant	P06731
CEACAM-1	P13688
CEACAM-3	P40198
Cerberus 1	O95813
Chemerin	Q99969
CHI3L1	P36222
CHL-1	O00533
CHMP2B	Q9UQN3
CHST1	O43916
CHST2	Q9Y4C5
CHST3	Q7LGC8
CHST4	Q8NCG5
cIAP-1	Q13490
cIAP-2	Q13489
CILP-1	O75339
Ck beta 8-1	P55773
CLEC-1	Q8NC01
CLEC10A	Q8IUN9
CLEC-2	Q9P126
CLEC9A	Q6UXN8
Clusterin	P10909
CMG-2	P58335
c-Myc	P01106
CNTF	P26441
CNTF R alpha	P26992
Coagulation Factor III	P13726
Coagulation Factor VII	P08709
Coagulation Factor X	P00742
Coagulation Factor XIV	P04070
Cochlin	O43405
Collectin-12	Q5KU26
Common beta Chain	P32927
Complement MASP3	P48740
COMT	P21964
Contactin-1	Q12860
Contactin-2	Q02246
Contactin-3	Q9P232
Contactin-5	O94779
Cortactin	Q14247
CREG	O75629
CRELD2	Q6UXH1
CRIM1	Q9NZV1

Cripto-1	P13385
CRISP-2	P16562
CrkL	P46109
CRP	P02741
CRTAC1	Q9NQ79
CRTAM	O95727
CTACK	Q9Y4X3
CTLA-4	P16410
CTRC	Q99895
CXADR	P78310
CXCL14	O95715
CXCL16	Q9H2A7
CXCL5	P42830
Cyclophilin A	P62937
Cyr61	O00622
Cystatin A	P01040
Cystatin B	P04080
Cystatin C	P01034
Cystatin D	P28325
Cystatin E/M	Q15828
Cystatin S	P01036
Cystatin SA	P09228
Cystatin SN	P01037
Cytokeratin 18	P05783
Cytokeratin 19	P08727
Cytokeratin 8	P05787
DAN	O95453
DAPP1	Q9UN19
DBH	P09172
DcR3	O95407
DC-SIGNR	Q9H2X3
DDR1	Q08345
Decorin	P07585
Dectin-1	Q9BXN2
Dectin-2	Q6EIG7
Desmin	P17661
Desmocollin-3	Q14574
Desmoglein-1	Q02413
Desmoglein-2	Q14126
Desmoglein-3	P32926
DFF45	O00273
DKK-1	O94907
DKK-3	Q9UBP4
DKK-4	Q9UBT3
DLL1	O00548
DLL4	Q9NR61
DNAM-1	Q15762

DNMT3A	Q9Y6K1
Dopa Decarboxylase	P20711
DPPII	Q9UHL4
DR3	Q93038
DR6	O75509
DRAK1	Q9UEE5
Draxin	Q8NBI3
DSCAM	O60469
DSPG3	Q99645
Dtk	Q06418
Dynactin subunit 1	Q14203
E-Cadherin	P12830
EDA-A2	Q92838
EDAR	Q9UNE0
EDIL3	O43854
EGF	P01133
EGFR	P00533
EG-VEGF	P58294
EMR2	Q9UHX3
Endocan	Q9NQ30
Endoglin	P17813
Endoglycan	Q9NZ53
eNOS	P29474
ENPP-2	Q13822
ENPP-7	Q6UWV6
Enteropeptidase	P98073
Eotaxin-1	P51671
Eotaxin-2	O00175
Eotaxin-3	Q9Y258
EphA1	P21709
EphA2	P29317
EphB2	P29323
EphB3	P54753
EphB4	P54760
EphB6	O15197
Ephrin-A4	P52798
Ephrin-B3	Q15768
Epimorphin	P32856
Epiregulin	O14944
ErbB2	P04626
ErbB3	P21860
ErbB4	Q15303
Erythropoietin	P01588
Erythropoietin R	P19235
ESAM	Q96AP7
E-Selectin	P16581
Exostosin-like 3	O43909

FABP1	P07148
FABP2	P12104
FABP4	P15090
FABP6	P51161
FABP8	P02689
FAM3C	Q92520
FAP	Q12884
Fas	P25445
Fas Ligand	P48023
Fc epsilon RI	P12319
Fc gamma RIIB/C	P31995/P31994
FCAR	P24071
FCRL1	Q96LA6
FCRL2	Q96LA5
FCRL3	Q96P31
FCRL5	Q96RD9
FCRLB	Q6BAA4
Ferritin	P02794
Fetuin A	P02765
FGF R5	Q8N441
FGF-12	P61328
FGF-16	O43320
FGF-17	O60258
FGF-19	O95750
FGF-20	Q9NP95
FGF-21	Q9NSA1
FGF-23	Q9GZV9
FGF-3	P11487
FGF-4	P08620
FGF-5	P12034
FGF-6	P10767
FGF-7	P21781
FGF-9	P31371
Fgr	P09769
Ficolin-1	O00602
FKBP51	Q13451
FLRG	O95633
FLRT1	Q9NZU1
FLRT2	O43155
Flt-3	P36888
Flt-3 Ligand	P49771
FOLH1	Q04609
Follistatin	P19883
Follistatin-like 1	Q12841
FOLR1	P15328
FOLR2	P14207
FosB	P53539

Fractalkine	P78423
FRS2	Q8WU20
FSH	P01215/P01225
FUCA1	P04066
Fucosyltransferase 8	Q9BYC5
Furin	P09958
Galanin	P22466
Galectin-1	P09382
Galectin-2	P05162
Galectin-3	P17931
Galectin-4	P56470
Galectin-7	P47929
Galectin-8	O00214
Galectin-9	O00182
GALNT10	Q86SR1
GALNT2	Q10471
GALNT3	Q14435
Gas 1	P54826
Gas 6	Q14393
GASP-1	Q5JY77
GASP-2	Q96D09
Gastric Intrinsic Factor	P27352
Gastrokine-1	Q9NS71
GATA-4	P43694
GATA-5	Q9BWX5
GBA3	Q9H227
GCP-2	P80162
GCSF	P09919
GCSF R	Q99062
GDF-11	O95390
GDF-15	Q99988
GDF-3	Q9NR23
GDF-8	O14793
GDF-9	O60383
GDNF	P39905
GFAP	P14136
GFR alpha-1	P56159
GFR alpha-2	O00451
GITR	Q9Y5U5
GITR Ligand	Q9UNG2
GLI-3	P10071
GLP-1	P01275
Glyoxalase 2	Q16775
Glypican 1	P35052
Glypican 2	Q8N158
Glypican 3	P51654
Glypican 5	P78333

GM-CSF	P04141
GM-CSF R alpha	P15509
GOLM1	Q8NBJ4
gp130	P40189
GPR111	Q8IZF7
GPR115	Q8IZF3
GPR56	Q9Y653
GPV	P40197
GPVI	Q9HCN6
Granulysin	P22749
Granzyme A	P12544
Granzyme B	P10144
Granzyme H	P20718
GRAP2	O75791
GRK5	P34947
GRO alpha	P09341
GRO alpha/beta/gamma	P19876/P09341/P19875
Growth Hormone	P01241
Growth Hormone R	P10912
GSTM1	P09488
HAI-1	O43278
HAI-2	O43291
HAPLN1	P10915
HB-EGF	Q99075
HCC-1	Q16627
HCC-4	O15467
hCG beta	P0DN86/P0DN87
HE4	Q14508
HepaCAM	Q14CZ8
Hepsin	P05981
HGF	P14210
HGFR	P08581
HIF-1 alpha	Q16665
HS3ST1	O14792
HS3ST3B1	Q9Y662
HS3ST4	Q9Y661
HSP32	P09601
htPAPP-A	Q13219
HTRA2	O43464
HVEM	Q92956
Hydroxyacid oxidase 1	Q9UJM8
I-309	P22362
ICAM-1	P05362
ICAM-2	P13598
ICAM-3	P32942
ICOS	Q9Y6W8
IDO	P14902

IFN-alpha/beta R2	P48551
IFN-beta	P01574
IFN-gamma	P01579
IFN-gamma R1	P15260
IGF-1	P05019
IGF-1 R	P08069
IGF-2	P01344
IGF-2 R	P11717
IGFBP-1	P08833
IGFBP-2	P18065
IGFBP-3	P17936
IGFBP-4	P22692
IGFBP-5	P24593
IGFBP-6	P24592
IGSF3	O75054
IGSF4B	Q8N126
IL-1 alpha	P01583
IL-1 beta	P01584
IL-1 R1	P14778
IL-1 R2	P27930
IL-1 R3	Q9NPH3
IL-1 R4	Q01638
IL-1 R6	Q9HB29
IL-1 Ra	P18510
IL-10	P22301
IL-10 R alpha	Q13651
IL-10 R beta	Q08334
IL-11	P20809
IL-12 p40	P29460
IL-12 p70	P29459/P29460
IL-13	P35225
IL-13 R alpha 1	P78552
IL-13 R alpha 2	Q14627
IL-15	P40933
IL-15 R alpha	Q13261
IL-16	Q14005
IL-17 RA	Q96F46
IL-17 RB	Q9NRM6
IL-17 RC	Q8NAC3
IL-17 RD	Q8NFM7
IL-17A	Q16552
IL-17B	Q9UHF5
IL-17C	Q9P0M4
IL-17E	Q9H293
IL-17F	Q96PD4
IL-18	Q14116
IL-18 BP alpha	O95998

IL-18 R alpha	Q13478
IL-18 R beta	O95256
IL-19	Q9UHD0
IL-2	P60568
IL-2 R alpha	P01589
IL-2 R beta	P14784
IL-2 R gamma	P31785
IL-20	Q9NYY1
IL-20 R alpha	Q9UHF4
IL-20 R beta	Q6UXL0
IL-21	Q9HBE4
IL-21 R	Q9HBE5
IL-22	Q9GZX6
IL-22 R alpha 1	Q8N6P7
IL-22BP	Q969J5
IL-23	P29460/Q9NPF7
IL-23 R	Q5VWK5
IL-24	Q13007
IL-27	Q8NEV9
IL-27 R alpha	Q6UWB1
IL-28 R alpha	Q8IU57
IL-28A	Q8IZJ0
IL-29	Q8IU54
IL-3	P08700
IL-31	Q6EBC2
IL-31 RA	Q8NI17
IL-32 alpha	P24001
IL-33	O95760
IL-34	Q6ZMJ4
IL-36 alpha	Q9UHA7
IL-36 beta	Q9NZH7
IL-36 gamma	Q9NZH8
IL-36 Ra	Q9UBH0
IL-37	Q9NZH6
IL-38	Q8WWZ1
IL-4	P05112
IL-4 R alpha	P24394
IL-5	P05113
IL-5 R alpha	Q01344
IL-6	P05231
IL-6 R	P08887
IL-7	P13232
IL-7 R alpha	P16871
IL-8	P10145
IL-9	P15248
ILT-2	Q8NHL6
ILT-4	Q8N423

Inhibin A	P05111/P08476
Insulin	P01308
Insulin R	P06213
Integrin alpha-1	P56199
Integrin alpha-2	P17301
Integrin alpha-5	P08648
Integrin alpha-M	P11215
IP-10	P02778
I-TAC	O14625
Jagged 1	P78504
Jagged 2	Q9Y219
JAM-A	Q9Y624
JAM-B	P57087
JAM-C	Q9BX67
Kallikrein 1	P06870
Kallikrein 11	Q9UBX7
Kallikrein 12	Q9UKR0
Kallikrein 14	Q9P0G3
Kallikrein 5	Q9Y337
Kallikrein 7	P49862
Kell	P23276
KIR2DL3	P43628
KIRREL3	Q8IZU9
KLF4	O43474
Kremen-2	Q8NCW0
Kynureninase	Q16719
LAG-3	P18627
LAIR1	Q6GTX8
LAIR2	Q6ISS4
Laminin alpha 4	Q16363
LAMP	Q13449
LAMP1	P11279
LAMP2	P13473
Langerin	Q9UJ71
LAP/TGF beta 1	P01137
Layilin	Q6UX15
LDL R	P01130
LEDGF	O75475
Legumain	Q99538
Leptin	P41159
Leptin R	P48357
LIF	P15018
LIF R alpha	P42702
LIGHT	O43557
LILRB4	Q8NHJ6
LIMPII	Q14108
Lin-28A	Q9H9Z2

Lipocalin-1	P31025
Lipocalin-2	P80188
LOX-1	P78380
LOXL2	Q9Y4K0
LRIG1	Q96JA1
LRIG3	Q6UXM1
LRP-6	O75581
LRRC4	Q9HBW1
LRRTM3	Q86VH5
LRRTM4	Q86VH4
LSECtin	Q6UXB4
L-Selectin	P14151
Lumican	P51884
Luteinizing hormone	P01215/P01229
Lymphotactin	P47992
Lymphotoxin beta R	P36941
LYVE-1	Q9Y5Y7
MAP1D	Q6UB28
Marapsin	Q9BQR3
Matrilin-2	O00339
Matrilin-3	O15232
Matriptase	Q9Y5Y6
MBL	P11226
MCEMP1	Q8IX19
Mcl-1	Q07820
MCP-1	P13500
MCP-2	P80075
MCP-3	P80098
MCP-4	Q99616
M-CSF	P09603
M-CSF R	P07333
MDC	O00626
MDGA1	Q8NFP4
MDGA2	Q7Z553
MDL-1	Q9NY25
MDM2	Q00987
MEF2C	Q06413
MEPE	Q9NQ76
Meprin alpha	Q16819
Meprin beta	Q16820
Mer	Q12866
Mesothelin	Q13421
METAP2	P50579
MFRP	Q9BY79
MIA	Q16674
MICA	Q29983
MICB	Q29980

Midkine	P21741
MIF	P14174
MIG	Q07325
MIP-1 alpha	P10147
MIP-1 beta	P13236
MIP-1 delta	Q16663
MIP-3 alpha	P78556
MIP-3 beta	Q99731
MIS RII	Q16671
MMP-1	P03956
MMP-10	P09238
MMP-12	P39900
MMP-13	P45452
MMP-2	P08253
MMP-3	P08254
MMP-7	P09237
MMP-8	P22894
MMP-9	P14780
MOG	Q16653
MPIF-1	P55773
MSP alpha/beta	P26927
MSP Receptor	Q04912
NAP-2	P02775
N-Cadherin	P19022
NCAM-1	P13591
NCK1	P16333
Nectin-1	Q15223
Nectin-2	Q92692
Nectin-3	Q9NQS3
Nectin-4	Q96NY8
Neprilysin	P08473
Neprilysin-2	Q495T6
Nesfatin-1	P80303
Nestin	P48681
Netrin-4	Q9HB63
Neudesin	Q9UMX5
Neurexin-3 beta	Q9HDB5
Neurocan	O14594
Neuroglycan C	O95196
Neurogranin	Q92686
Neuroligin 2	Q8NFZ4
Neuropilin-2	O60462
Neurturin	Q99748
NG2	Q6UVK1
NGFR	P08138
Nidogen-1	P14543
Nidogen-2	Q14112

NKp30	O14931
NKp44	O95944
NKp46	O76036
NKp80	Q9NZS2
Nogo Receptor	Q9BZR6
Nogo-A	Q9NQC3
Norrin	Q00604
Notch-1	P46531
Notch-3	Q9UM47
NOV	P48745
NPDC-1	Q9NQX5
NPTXR	O95502
NQO-1	P15559
Nr-CAM	Q92823
Nrf2	Q16236
NRG1-alpha	Q02297
NRG1-beta 1	Q02297
NSE	P09104
NT-3	P20783
NT-4	P34130
NTAL	Q9GZY6
NTB-A	Q96DU3
NUDT5	Q9UKK9
Numb	P49757
NUP85	Q9BW27
Olfactomedin-2	O95897
Olig2	Q13516
Omgp	P23515
Oncostatin M	P13725
Oncostatin M R beta	Q99650
OSCAR	Q8IYS5
Osteoactivin	Q14956
Osteoadherin	Q99983
Osteopontin	P10451
Osteoprotegerin	O00300
OX40	P43489
OX40 Ligand	P23510
p27/Kip1	P46527
p53	P04637
p63	Q9H3D4
PAI-1	P05121
Pancreatic Polypeptide	P01298
PAPP-A	Q13219
PAPP-A2	Q9BXP8
PAR1	P25116
PARC	P55774
Pax3	P23760

P-Cadherin	P22223
PCK1	P35558
PCSK2	P16519
PD-1	Q15116
PD-ECGF	P19971
PDGF R alpha	P16234
PDGF R beta	P09619
PDGF-AA	P04085
PDGF-AB	P01127/P04085
PDGF-BB	P01127
PDGF-C	Q9NRA1
PD-L2	Q9BQ51
PDX-1	P52945
PEAR1	Q5VY43
PECAM-1	P16284
Pentraxin-3	P26022
Pepsinogen 1	P0DJD8/P0DJD7/P0DJD9
Pepsinogen 2	P20142
Periostin	Q15063
Peroxiredoxin-2	P32119
Peroxiredoxin-4	Q13162
Persephin	O60542
PGRP-S	O75594
PILR-alpha	Q9UKJ1
Platelet Factor 4	P02776
Plexin A4	Q9HCM2
Plexin B3	Q9ULL4
Plexin D1	Q9Y4D7
PLGF	P49763
PlGF-2	P49763
Podocalyxin	O00592
Podoplanin	Q86YL7
POGLUT1	Q8NBL1
PON1	P27169
Pref-1	P80370
PRELP	P51888
Presenilin 1	P49768
Procalcitonin	P01258
Progranulin	P28799
ProGRP	P07492
Prolactin	P01236
Prolactin R	P16471
Prostasin	Q16651
PSA-free	P07288
PSA-total	P07288
PSMA1	P25786
PTH	P01270

PTH1R	Q03431
PTP1B	P18031
PU.1	P17947
PYY	P10082
RAGE	Q9UQ07
RalA	P11233
RANK	Q9Y6Q6
RANTES	P13501
RBP4	P02753
RCOR1	Q9UKL0
Reg1B	P48304
Reg3A	Q06141
Reg4	Q9BYZ8
RELT	Q969Z4
Renin	P00797
Resistin	Q9HD89
Ret	P07949
RGM-A	Q96B86
RGM-B	Q6NW40
RGM-C	Q6ZVN8
ROBO2	Q9HCK4
ROBO3	Q96MS0
ROBO4	Q8WZ75
ROR1	Q01973
ROR2	Q01974
R-Spondin 2	Q6UXX9
Ryk	P34925
S100 A1	P23297
S100 A13	Q99584
S100 A8	P05109
SALM4	Q9BTN0
SCCA2	P48594
SCF	P21583
SCF R	P10721
SDF-1 alpha	P48061
SDF-1 beta	P48061
Semaphorin 4C	Q9C0C4
Semaphorin 4D	Q92854
Semaphorin 4G	Q9NTN9
Semaphorin 6A	Q9H2E6
Semaphorin 6B	Q9H3T3
Semaphorin 6C	Q9H3T2
Semaphorin 6D	Q8NFY4
Semaphorin 7A	O75326
SERPIN A4	P29622
SERPIN A5	P05154
Serpin B6	P35237

Serpin B8	P50452
SERPIN F1	P36955
sFRP-3	Q92765
SH2D1A	O60880
SHP-1	P29350
SIGIRR	Q6IA17
Siglec-1	Q9BZZ2
Siglec-10	Q96LC7
Siglec-11	Q96RL6
Siglec-2	P20273
Siglec-5	O15389
Siglec-6	O43699
Siglec-7	Q9Y286
Siglec-9	Q9Y336
SIRP alpha	P78324
SIRP-gamma	Q9P1W8
Sirtuin 1	Q96EB6
Sirtuin 2	Q8IXJ6
Sirtuin 5	Q9NXA8
SLAM	Q13291
SLITRK5	O94991
Smad4	Q13485
SMPD1	P17405
Sonic Hedgehog N-Terminal	Q15465
SorCS1	Q8WY21
SorCS2	Q96PQ0
SorCS3	Q9UPU3
Sortilin	Q99523
SOST	Q9BQB4
SOX15	O60248
SOX2	P48431
SOX7	Q9BT81
SOX9	P48436
SP-D	P35247
SPHK1	Q9NYA1
Spinesin	Q9H3S3
SPINK1	P00995
SR-AI	P21757
SREC-I	Q14162
SREC-II	Q96GP6
ST3GAL1	Q11201
ST6GAL1	P15907
ST8SIA1	Q92185
Stabilin-2	Q8WWQ8
SULT2A1	Q06520
Syndecan-1	P18827
Syndecan-3	O75056

Syndecan-4	P31431
Syntaxin 4	Q12846
Syntaxin 6	O43752
TACE	P78536
TACI	O14836
TAFA1	Q7Z5A9
TAFA2	Q8N3H0
TAFA5	Q7Z5A7
TARC	Q92583
TAZ	Q9GZV5
TC-PTP	P17706
TECK	O15444
Tenascin R	Q92752
Testican 2	Q92563
TFF3	Q07654
TFPI	P10646
TFPI-2	P48307
TGF alpha	P01135
TGF beta 1	P01137
TGF beta 2	P61812
TGF beta 3	P10600
TGF beta R3	Q03167
THAP11	Q96EK4
Thrombomodulin	P07204
Thrombopoietin	P40225
Thrombospondin-1	P07996
Thrombospondin-2	P35442
Thrombospondin-5	P49747
THSD1	Q9NS62
Thyroglobulin	P01266
Thyroid Peroxidase	P07202
Tie-1	P35590
Tie-2	Q02763
TIM-1	Q96D42
TIM-3	Q8TDQ0
TIMP-1	P01033
TIMP-2	P16035
TIMP-4	Q99727
TLR1	Q15399
TLR2	O60603
TLR3	O15455
TLR4	O00206
TMEFF1	Q8IYR6
TNF alpha	P01375
TNF beta	P01374
TNF RI	P19438
TNF RII	P20333

TPST2	O60704
TRACP	P13686
TRAIL	P50591
TRAIL R1	O00220
TRAIL R2	O14763
TRAIL R3	O14798
TRAIL R4	Q9UBN6
TRANCE	O14788
Transcobalamin II	P20062
Transferrin	P02787
Transglutaminase 3	Q08188
Transglutaminase 4	P49221
Trappin-2	P19957
TREM-1	Q9NP99
TREM-2	Q9NZC2
TREML1	Q86YW5
Tripeptidyl-peptidase I	O14773
TrkA	P04629
TRKC	Q16288
TROP1	P16422
TROP-2	P09758
Troponin C	P63316
Troponin I	P48788
TROY	Q9NS68
Trypsin 1	P07477
Trypsin 3	P35030
TSH	P01215/P01222
TSLP	Q969D9
TSLP R	Q9HC73
Tsukushi	Q8WUA8
TWEAK	O43508
TWEAK R	Q9NP84
Ubiquitin+1	P0CG47
UCH-L3	P15374
ULBP-1	Q9BZM6
ULBP-2	Q9BZM5
ULBP-3	Q9BZM4
ULBP-4	Q8TD07
UNC5H3	O95185
UNC5H4	Q6UXZ4
uPA	P00749
uPAR	Q03405
Uromodulin	P07911
VAMP-1	P23763
VAMP-2	P63027
VAP-A	Q9P0L0
VCAM-1	P19320

VE-Cadherin	P33151
VEGF-A	P15692
VEGF-C	P49767
VEGF-D	O43915
VEGFR1	P17948
VEGFR2	P35968
VEGFR3	P35916
Visfatin	P43490
vWF-A2	P04275
WIF-1	Q9Y5W5
WISP-1	O95388
Wnt-4	P56705
XEDAR	Q9HAV5
XIAP	P98170

Table S4: Average group concentrations of each protein assessed in the 21 matched serum, DBS, and DSSs including the assay detection ranges for each protein. Data are expressed as average ± SE. ND: not detectable (below the LOD in at least 2/3 of the samples for each group)

Biomarker	DBS (pg/ml)	DSS (pg/ml)	Serum (pg/ml)	LOD (pg/ml)	Max (pg/ml)
BLC	118.4±34.6	157.8±45	16.22±4.92	0.16	74.07
4-1BB	2775.2±1087.6	5487.6±2259.8	1092.5±420.24	0.17	148.15
6Ckine	326764.8±32827.8	583661.4±66867.2	19097.58±3892.62	0.55	333.33
Activin A	3623±467.8	8118.4±508	238.7±39.28	1.41	2222.22
AgRP	2877.6±360	6762±617.4	182.52±34.32	8.21	1000.00
ALCAM	9651.6±492.6	14787.6±945	1953.88±145.08	0.55	1333.33
ANG-1	56934.6±5050.8	52356.4±6233.6	2411.8±238.48	5.30	11111.11
Angiogenin	8088±1589	14918.8±414.8	1624.34±59.26	83.90	2000.00
Angiostatin	1499713.2±96125.2	1152884.6±60610.2	34596.56±4216.32	8.75	2000.00
AR	1310.2±254.4	1562.4±324.2	171.94±44.38	0.11	111.11
Axl	1449.8±135.6	2907.2±229.8	164.9±22.1	24.57	2000.00
B7-1	3031.4±573.4	3131±740.2	424.26±115.82	1.50	2000.00
BCMA	18365±1210.2	26322.6±2148.4	3994.92±446.66	2.24	222.22
BDNF	1042.8±161	881.8±152.4	151.74±29.68	17.78	4000.00
bFGF	ND	354.2±34.4	19.32±3.64	2.40	2000.00
BMP-4	ND	ND	ND	1.10	3333.33
BMP-5	178287.4±22606.2	305352.2±23861.4	16734.86±2551.72	3.91	4000.00
BMP-7	ND	39232.2±7211.4	4735.18±1233.46	0.52	500.00
b-NGF	687.6±199	758.8±169.6	84.96±18.24	0.45	444.44
BTC	1378.8±144.8	1896.2±211.6	126.88±20.04	4.32	2222.22
Cathepsin S	30359.4±4725	24406.2±3906.4	2362.56±636.5	0.66	10000.00
CCL28	17073.8±4460	26964±6460.6	3113.36±1000.36	0.24	500.00
CD14	102459.6±4769	98207.6±3704.4	10645.92±536.42	1.12	111.11
CD30	3382±757	4680.2±740	ND	4.95	4000.00
CD40	2108.6±173.4	4204.6±413.6	120.98±25.2	6.35	5000.00
CD40L	643.4±76	679.6±74.4	26.66±5.02	0.36	444.44
CEACAM-1	5269.6±646	4667.8±522.8	249.64±33.94	5.90	74.07
Contactin-2	4976.2±992	6915±1330.2	ND	0.34	1333.33
Cripto-1	1541.2±206.2	1797.6±216.8	157.16±32.32	3.27	5000.00
CTACK	36821.6±5305.4	53974.8±5479	ND	0.93	3333.33
CXCL16	12225.4±1251	25723.8±4222.6	3223.5±599.58	0.32	333.33
DAN	6130.4±846.4	12591.8±1473.8	503.96±111.16	0.30	10000.00
DKK-1	29988.2±2684	41666.2±3722.6	1505.9±280.72	1.59	2000.00
DR6	2998.8±196	4097±439.6	821.26±166	0.82	6666.67
Dtk	2523.8±624	2954.4±831.2	593.56±149.18	6.01	1481.48
E-Cadherin	23551.2±1649.8	29369.4±1923	987.94±189.02	1.30	4444.44
EGF	34±4	10.4±2.8	1.56±0.44	17.13	222.22
EGF R	35130.4±3540.8	41520.8±3931.4	6220.16±542.78	345.68	20000.00
EG-VEGF	791.8±146.6	817.4±145.2	86.14±16.24	6.52	4444.44

ENA-78	7849.2±1011.8	3866.6±633.6	185.28±32.72	1.59	4444.44
Endoglin	3273.4±385.2	1123.2±297	183.36±32.76	4.38	3333.33
Eotaxin	228.4±21.4	42±7.6	10.26±1.76	0.61	666.67
Eotaxin-2	272.2±51.2	205.8±32.6	63.12±10.34	2.36	2222.22
Eotaxin-3	6800.6±1646.2	23244.8±3368.8	1074.26±294.54	712.12	33333.33
EpCAM	3242.2±1126.8	3813.6±1071.6	372.34±116.88	1885.99	100000.00
ErbB3	1847.8±267.8	2682.8±434.2	403.14±55.88	181.07	40000.00
E-Selectin	1827.8±217.2	1436.6±194.8	558.38±77.54	1.43	3333.33
Fas	979.8±167.6	594.8±131	120.78±17.1	0.02	66.67
FAS L	2707.6±673.8	3233.2±647.6	165.1±39.74	1.24	10000.00
Fcg RIIBC	8721.4±973.6	12472.2±1738	212.68±37.56	0.48	10000.00
FGF-4	9551.8±1995	10132±1917	1222.72±233.48	48.82	11111.11
FGF-7	ND	2712.2±564.2	555.12±116.06	11.90	3333.33
Flt-3L	383.4±63	358.2±68	40.66±5.42	0.30	666.67
Follistatin	12169.4±865.4	28360.8±1931.4	757.44±144.2	2.86	4000.00
Galectin-7	124460±23649.8	90876±26108.4	8815.4±3102.06	1.89	1111.11
GCP-2	2429.6±283.2	2730±381.6	113.62±44.48	0.27	370.37
G-CSF	ND	ND	ND	3.14	4000.00
GDF-15	1671±140	2071.8±176	125.72±26.24	1.47	1666.67
GDNF	973±323.2	917.8±174.2	139.68±35.86	11.72	6666.67
GH	1203.6±263.8	1556.2±315.6	198.1±44	97.31	200000.00
GITR	7165.8±1725.4	ND	1405±384.44	316.22	200000.00
GM-CSF	ND	ND	ND	77.53	33333.33
gp130	68295±4384.6	100372.8±7919.6	7429.8±762.06	33.96	20000.00
GRO	ND	ND	ND	12.89	20000.00
HB-EGF	304.8±69.6	415.4±122.8	31.96±10.42	7.92	13333.33
HCC-1	29595.2±1956.4	31005.4±2238.2	2670.7±222.74	1.02	1111.11
HCC-4	5800.6±597.4	9543.4±1002.8	924.32±76	6.86	13333.33
HGF	1108.2±166	928.6±121.8	119.54±29.38	2.79	3333.33
HVEM	4040.6±673	2776.6±656.2	414.94±104.48	0.85	4000.00
I-309	ND	ND	ND	2.27	3333.33
ICAM-1	47393.6±3969.4	60154.4±6585.6	9630.04±1033.94	0.52	1333.33
ICAM-2	165066.8±38112.6	205532.8±32617.6	20210.04±4364.6 8	1.56	1111.11
ICAM-3	5901±1296	6833.2±1383	624.54±142.28	3.87	20000.00
IFNg	ND	ND	ND	0.96	370.37
IGF-1	ND	ND	1142.04±358.4	2500.60	100000.00
IGFBP-1	9631.4±1409.6	6656.4±1247.8	938.74±172.02	16.82	13333.33
IGFBP-2	135355.4±9756.4	141986.4±10113	18638.78±1128.7 4	0.69	3333.33
IGFBP-3	220318.2±22497.6	319510.8±33863.8	5915.14±747.84	11.61	10000.00
IGFBP-4	381706.8±31651	828325.4±72184.2	38567.98±6041.6 2	56.45	40000.00

IGFBP-6	293110.6±31576.2	393001.6±34289	19586.02±2173.86	6.13	20000.00
IL-1 R4	365.4±87.6	640.2±120.2	53.66±10.34	664.15	40000.00
IL-1 RI	317.8±81	277±68.6	47.82±14.78	3.85	444.44
IL-10	ND	ND	ND	7.95	2222.22
IL-10 Rb	2379.8±577.4	2919.6±752.6	519.3±156.7	28.65	40000.00
IL-11	ND	ND	ND	381.51	50000.00
IL-12p40	152.8±22.8	207.8±27.8	57.24±10.32	4.06	6666.67
IL-12p70	ND	ND	ND	1.52	10000.00
IL-13	ND	55.2±8.2	ND	4.18	20000.00
IL-13 R1	15930.2±1450.4	24173.8±1872.8	1140.44±194.14	0.79	10000.00
IL-13 R2	6054.2±1355.2	10578.2±1670.4	1102.22±255.8	18.92	1000.00
IL-15	5259.4±1582.2	4552.2±1396.2	485.86±152.22	1.46	4000.00
IL-16	8849.6±1757	9662.4±2903.8	846.86±269.44	2.13	1111.11
IL-17	ND	ND	ND	141.95	7407.41
IL-17B	24306.2±1660.8	55485.4±3747.6	1690.56±302.32	17.38	100000.00
IL-17F	27666.8±7877	50419.6±21703.8	5374.32±2236.24	147.89	6666.67
IL-17R	33423±6075.8	33937.4±6384.4	5178.1±1300.96	15.41	10000.00
IL-18 BPa	37596.2±2977.4	42759±4635.2	2121.94±478.78	216.64	100000.00
IL-1a	ND	ND	ND	2.62	1481.48
IL-1b	33.2±6.4	ND	ND	1.42	3333.33
IL-1ra	2095.8±345.2	2225.6±412.4	ND	12.43	10000.00
IL-2	ND	ND	ND	18.20	13000.00
IL-2 Ra	3233.2±236.6	7566.2±468	230.42±32.64	10.23	10000.00
IL-2 Rb	40723±3360.6	107662.6±7875.6	4099.94±725.52	9.65	33333.33
IL-2 Rg	ND	ND	ND	1.16	222.22
IL-21R	18053.6±3647.8	ND	2966.06±858.12	0.33	1333.33
IL-23	65194±7905.8	115356.8±9350.4	4588.06±902.14	0.30	370.37
IL-28A	4142.6±513.4	6650.4±715.2	137.16±19.2	0.84	3333.33
IL-29	178250±17998.2	225489.8±24214.4	ND	14.60	4000.00
IL-31	2636.4±899.2	6699±2572.8	754.88±309.66	0.19	444.44
IL-4	ND	ND	ND	5.08	6666.67
IL-5	ND	931.6±167.6	ND	14.21	10000.00
IL-6	ND	ND	ND	7.79	11111.11
IL-6R	19653.6±1327.6	23736.6±1151	2691.82±133.62	0.58	148.15
IL-7	ND	ND	ND	22.42	100000.00
IL-8	ND	ND	ND	0.33	148.15
IL-9	15760.8±4446.2	24172.6±3674.2	ND	552.84	33333.33
Insulin	3587.6±1276	3483±561.8	420.56±110.34	2.23	1111.11
IP-10	773.4±64.6	1413.6±134.4	59.16±9.12	0.76	1111.11
I-TAC	13378.6±2207	15848.8±1994.4	ND	7.50	3703.70
LAP(TGFb1)	17712.6±3031.2	16403.4±1482.4	507.88±121.48	1.11	370.37
LIF	21637.8±1762.4	31078.6±1760.6	ND	1.27	3333.33

LIGHT	8133±1020.6	9012.2±1137.2	388±108.66	6.50	10000.00
LIMPII	3284.6±929.6	5139.8±1888.6	1437.14±585.5	6.27	10000.00
Lipocalin-2	22636.6±1110.6	11372±602.2	495.76±46.56	1.86	6666.67
L-Selectin	304271.2±23760.4	329852.6±21105.4	48528.38±4651.8	5.70	3333.33
Lymphotactin	5700±525.6	12844.8±1261.4	358.14±113.92	20.12	3333.33
LYVE-1	13229.4±823	17152.2±910.6	1714.04±149.1	0.38	1111.11
MCP-1	382±51.2	414.8±60	ND	7.88	10000.00
MCP-2	1214.4±252.4	2103.6±400.6	68.44±20.96	0.24	4000.00
MCP-3	128.6±21	405.8±49.2	10.9±3.12	4.43	6666.67
MCP-4	65.4±10.2	143.4±17.2	7.94±2.18	4.08	4000.00
MCSF	ND	64.2±19.6	ND	4.16	20000.00
MCSF R	65461.6±9473	76852.8±7912.8	9191.58±896.94	7.59	4444.44
MDC	4463.2±977.4	5157±1022.4	134.86±28.4	1.43	2000.00
MICA	2480±678.4	1564±669	216.76±61.78	0.64	2000.00
MICB	83488.8±21529.8	104516.6±33182.8	16774.38±4876.64	38.34	10000.00
MIF	853722.4±50130.6	53773.2±5242.8	703.42±111.02	10.51	13333.33
MIG	473.8±116.8	889±231.2	102.94±31.72	7.29	11111.11
MIP-1a	905.2±272.4	1821.4±837.4	230.6±89.18	20.40	33333.33
MIP-1b	84.4±15.4	43.6±8	ND	0.94	1333.33
MIP-1d	4697.8±927.6	6870.6±1096.4	1328.94±271.22	0.32	1111.11
MIP-3a	125±19.4	215.2±19.2	4.38±1.12	3.18	4000.00
MIP-3b	4129.8±1047	9539.8±3188	1178.82±388.7	169.59	10000.00
MPIF-1	3696.6±491.4	4610.6±425.8	377.34±49.64	35.32	20000.00
MSP	12414.6±2076	16153.8±3775.6	3216.9±547.72	2.80	4000.00
NAP-2	6500±780.6	6273.6±487.2	627.5±56.92	0.49	1000.00
NGF R	722±151.2	720.6±114.6	85.36±20.22	118.34	3703.70
NrCAM	7459.2±730.6	10901.8±1148.4	353.32±63.82	0.40	2000.00
NRG1-b1	519±134.4	ND	ND	3.18	10000.00
NT-3	4297.8±1010.8	3737.6±646.4	498.56±129.76	49.09	15000.00
NT-4	2877.2±742.8	3793.8±982.2	388.18±89.56	3.88	15000.00
OPG	694.4±81.2	524.2±47.6	28.68±5.02	104.46	33333.33
OPN	230268±21709.2	272067±24602.4	2005.16±342.54	40.25	6666.67
PAI-1	44789±3614.6	48114±2924.8	4152.44±313.34	0.93	3333.33
PARC	827.6±83.6	834±64.6	58.16±3.36	1.13	10000.00
PDGF Rb	14826.4±5274.2	17162±3708.4	1557.48±243.58	2.98	5000.00
PDGF-AA	14821.4±1372.2	14761.4±2157.6	1940.8±233.74	2.41	3333.33
PDGF-AB	19223.6±1171.6	18967.4±1315.2	1213.38±136.46	28.68	40000.00
PDGF-BB	69334.6±7870	53928.6±6654.6	8962.08±1256.2	88.18	66666.67
PECAM-1	29175.8±2220	9635.6±1786.8	696.52±130.06	14.97	10000.00
PF4	515754.6±30548.2	522313.6±30407.4	46740.16±2574.54	5.10	3703.70

PIGF	374.6±73.6	311±48	22.22±5.7	7.39	3333.33
RAGE	4558.4±340.6	3886.2±358.8	546.14±56.98	1.01	666.67
RANTES	50182.8±1669.6	42881.6±2742.8	4317.76±257.08	10.19	13333.33
Resistin	53445.8±2768.2	5553.4±559.6	459.2±69.76	1074.49	1000000.00
SCF	637.8±131.2	661.4±133.8	45.02±10.86	3.32	3333.33
SCF R	38883.6±2238.2	46443.2±3248.6	6181.48±418.38	4.78	10000.00
SDF-1a	2331.6±295.2	5506±587.2	207.98±61.32	1.18	3333.33
SDF-1b	4275.6±604	6193±790	396.4±75.4	15.53	13333.33
Shh-N	2298.2±257	4281±416.2	181.42±29.14	10.69	26666.67
Siglec-5	17169.8±2652.6	18506±2829.2	2638.7±538.6	17.46	26666.67
TARC	667.4±77	812.6±75.8	37.6±6.86	2.78	6666.67
TECK	3534.4±497.4	10157.6±1208.8	211.1±49.44	1.02	666.67
TGFa	3990±1458.6	6059.2±2365	741.14±257.02	2.40	10000.00
TGFb1	454124.6±73015.6	793722.4±172669	46155.06±13403.6	30.15	13333.33
TGFb2	21059±1799.4	53177.4±3430	1419.38±222.88	33.75	33333.33
TGFb3	6307.4±1432.4	9386.4±2462	ND	24.50	33333.33
Tie-2	14138.6±1336.8	30170±1727.8	725.22±91.04	32.01	3333.33
TIM-1	1925.2±642.2	3599±1398.4	859.24±369.3	7.18	6666.67
TIMP-1	54015.4±2247.6	55799.8±1991.6	3714.4±245.92	46.41	13333.33
TIMP-2	52157.2±1751	54507.6±1864	6842.94±231.28	5.74	10000.00
TNF RI	2488.2±198	4042.2±345	971.76±87.96	83.70	33333.33
TNF RII	17143±646.8	22185.2±1416.8	3082.18±237.52	131.54	40000.00
TNFa	ND	1247.8±255.8	ND	8.10	4000.00
TNFb	ND	ND	ND	11.49	6666.67
TPO	21795±1575	46720.4±4067	1115.5±202.54	24.67	13333.33
TRAIL R3	7935.8±801	5151.8±514.4	417.58±51.28	5.06	3333.33
TRAIL R4	2330.8±254.4	4943.6±524.8	201.9±32.76	1.01	6666.67
Trappin-2	24826.8±1937.4	24180±1105.8	3066.78±272.96	1.95	1481.48
TREM-1	38684.8±3738.2	74753.8±5401.8	1471.16±206.54	135.43	26666.67
TSLP	1454±366.8	2783.4±905.8	504.74±271.82	5.14	13333.33
uPAR	18065.6±2466.4	7073.8±1427.2	905.2±167.08	6.68	3333.33
VCAM-1	219765.8±17533	367596.8±28526.2	48365.9±4276.06	0.37	1333.33
VEGF	378.4±53.2	329.2±46.8	ND	60.60	13333.33
VEGF R1	37459.4±6564.2	72694.6±10694.4	5169.42±1132.96	15.64	10000.00
VEGF R2	6480.4±647.2	10430.8±1104.2	929.8±114.96	29.73	22222.22
VEGF R3	24726.2±4921.4	30178.4±5631.8	4589.1±1002.14	1.99	2666.67
VEGF-C	7977.4±800.4	13755.2±1165.2	376±57.04	55.79	20000.00
VEGF-D	2364±690.2	2914.8±614	506.74±128.84	17.05	2222.22
XEDAR	ND	ND	367.5±114.92	59.75	40000.00

Dried Blood Sample Analysis by Antibody Array

Table S5: Passing-Bablok regression of each measurable protein in the 21 matched serum, DBS, and DSSs.

Biomarker	Y	X	Regression	constant	slope	Pearson's r
4-1BB	DSS	DBS	Passing-Bablok	-14.68939214	1.575364	0.967374774
6Ckine	DSS	DBS	Passing-Bablok	-27524.6229	3.411987	0.209727627
Activin A	DSS	DBS	Passing-Bablok	199.1443367	1.383496	0.23141271
AgRP	DSS	DBS	Passing-Bablok	-183.8167573	3.433659	0.297204306
ALCAM	DSS	DBS	Passing-Bablok	2178.704889	-3.07328	0.038176575
ANG-1	DSS	DBS	Passing-Bablok	-1272.140002	1.343087	0.276404609
Angiogenin	DSS	DBS	Passing-Bablok	738.5251047	0.08133	0.110766199
Angiostatin	DSS	DBS	Passing-Bablok	108154.3106	-0.64278	-0.477693194
AR	DSS	DBS	Passing-Bablok	4.384258065	1.434798	0.760588494
Axl	DSS	DBS	Passing-Bablok	-26.02461584	2.391228	0.292443798
B7-1	DSS	DBS	Passing-Bablok	-21.13462912	1.234376	0.860669893
BCMA	DSS	DBS	Passing-Bablok	-580.2572684	1.979832	0.615171019
BDNF	DSS	DBS	Passing-Bablok	0.370295053	0.8374	0.741637544
BLC	DSS	DBS	Passing-Bablok	0	1.340362	0.989762981
BMP-5	DSS	DBS	Passing-Bablok	8340.145709	0.966737	0.621925527
b-NGF	DSS	DBS	Passing-Bablok	1.999220346	0.932782	0.765912952
BTC	DSS	DBS	Passing-Bablok	-26.51174631	1.683231	0.239724672
Cathepsin S	DSS	DBS	Passing-Bablok	333.1000072	0.539067	0.890935806
CCL28	DSS	DBS	Passing-Bablok	89.3986444	1.638797	0.92034511
CD14	DSS	DBS	Passing-Bablok	1703.414003	0.635059	0.500740093
CD30	DSS	DBS	Passing-Bablok	71.22775552	1.537616	0.736156529
CD40	DSS	DBS	Passing-Bablok	-98.93705344	2.997912	0.53750227
CD40L	DSS	DBS	Passing-Bablok	4.398955732	0.913582	0.472364946
CEACAM-1	DSS	DBS	Passing-Bablok	-22.26920926	1.026198	0.71089131
Contactin-2	DSS	DBS	Passing-Bablok	0	1.489111	0.787241403
Cripto-1	DSS	DBS	Passing-Bablok	3.90106023	1.103094	0.728333309
CTACK	DSS	DBS	Passing-Bablok	3745.820466	-0.87167	0.104740827
CXCL16	DSS	DBS	Passing-Bablok	-306.3576459	2.504278	0.734336398
DAN	DSS	DBS	Passing-Bablok	92.79988713	1.920647	0.403017041
DKK-1	DSS	DBS	Passing-Bablok	14.62903605	1.562142	0.371456664
DR6	DSS	DBS	Passing-Bablok	-67.43288143	1.784132	0.725399964
Dtk	DSS	DBS	Passing-Bablok	-28.89756308	1.420676	0.951869963
E-Cadherin	DSS	DBS	Passing-Bablok	231.0602383	1.001674	0.283695641
EGF	DSS	DBS	Passing-Bablok	-0.145429844	0.310304	0.150673441
EGF R	DSS	DBS	Passing-Bablok	89.09940468	1.147667	0.71674045
EG-VEGF	DSS	DBS	Passing-Bablok	1.941182484	0.953396	0.772106465
ENA-78	DSS	DBS	Passing-Bablok	105.1915132	0.151934	0.532136616
Endoglin	DSS	DBS	Passing-Bablok	-29.50997181	0.462879	0.392176395
Eotaxin	DSS	DBS	Passing-Bablok	-0.028757579	0.14076	0.384594359
Eotaxin-2	DSS	DBS	Passing-Bablok	0.173826094	0.775292	0.890341806
Eotaxin-3	DSS	DBS	Passing-Bablok	-123.0843462	3.791493	0.745041225
EpCAM	DSS	DBS	Passing-Bablok	20.30020795	1.122945	0.962676379
ErbB3	DSS	DBS	Passing-Bablok	-38.67747253	1.943843	0.729036209
E-Selectin	DSS	DBS	Passing-Bablok	-7.300349492	0.881254	0.628616147
Fas	DSS	DBS	Passing-Bablok	-9.221853398	0.757843	0.947132363
FAS L	DSS	DBS	Passing-Bablok	32.66472551	1.019036	0.971001483
Fcg RIIBC	DSS	DBS	Passing-Bablok	-234.194075	1.928477	0.857183023
FGF-4	DSS	DBS	Passing-Bablok	-19.33750281	0.838161	0.703538103
Flt-3L	DSS	DBS	Passing-Bablok	-3.482836603	1.123411	0.887466555
Follistatin	DSS	DBS	Passing-Bablok	-910.3326248	3.863434	0.425036668
Galectin-7	DSS	DBS	Passing-Bablok	-1145.659607	0.99306	0.921372224
GCP-2	DSS	DBS	Passing-Bablok	378.4951437	-2.0593	0.261186076
GDF-15	DSS	DBS	Passing-Bablok	-10.76857585	1.310809	0.745477898
GDNF	DSS	DBS	Passing-Bablok	1.619739324	1.308038	0.728067983
GH	DSS	DBS	Passing-Bablok	9.195521271	1.062215	0.68188819
gp130	DSS	DBS	Passing-Bablok	-4999.67429	2.881828	0.48428538
HB-EGF	DSS	DBS	Passing-Bablok	0.159607226	1.338593	0.852556267
HCC-1	DSS	DBS	Passing-Bablok	4463.595738	-1.96152	-0.131331005
HCC-4	DSS	DBS	Passing-Bablok	-88.01968881	1.819972	0.202975084
HGF	DSS	DBS	Passing-Bablok	-4.526009906	0.884292	0.601965747
HVEM	DSS	DBS	Passing-Bablok	-67.30230944	0.935808	0.909969944
ICAM-1	DSS	DBS	Passing-Bablok	-441.1808555	1.455199	0.829226863
ICAM-2	DSS	DBS	Passing-Bablok	2783.133822	0.911831	0.937825875
ICAM-3	DSS	DBS	Passing-Bablok	-17.27248671	1.046867	0.931665109
IGFBP-1	DSS	DBS	Passing-Bablok	-18.90616203	0.618824	0.682905683
IGFBP-2	DSS	DBS	Passing-Bablok	27.24040008	0.976723	0.631583285
IGFBP-3	DSS	DBS	Passing-Bablok	76.92387433	1.483926	0.495025421
IGFBP-4	DSS	DBS	Passing-Bablok	-14777.10695	3.301881	0.34475247
IGFBP-6	DSS	DBS	Passing-Bablok	1242.058269	1.307284	0.467771684
IL-1 R4	DSS	DBS	Passing-Bablok	8.948093807	1.399087	0.777607412
IL-1 RI	DSS	DBS	Passing-Bablok	0.356243762	0.942639	0.824223282
IL-10 Rb	DSS	DBS	Passing-Bablok	-20.18313745	1.298354	0.960236935
IL-12p40	DSS	DBS	Passing-Bablok	1.132461015	1.477052	0.658323017
IL-13 R1	DSS	DBS	Passing-Bablok	-297.98987	1.935527	0.577248907
IL-13 R2	DSS	DBS	Passing-Bablok	150.4070605	1.173745	0.918092889
IL-15	DSS	DBS	Passing-Bablok	6.787068377	1.098886	0.979802453
IL-16	DSS	DBS	Passing-Bablok	-124.4271449	1.051747	0.949013079
IL-17B	DSS	DBS	Passing-Bablok	-1428.448207	3.371674	0.560429708
IL-17F	DSS	DBS	Passing-Bablok	-1332.721114	2.743007	0.97897812
IL-17R	DSS	DBS	Passing-Bablok	-325.0156218	1.054637	0.83319804
IL-18 BPa	DSS	DBS	Passing-Bablok	-1726.115569	1.93852	0.449080558
IL-1ra	DSS	DBS	Passing-Bablok	-17.34936162	1.020744	0.591556908
IL-2 Ra	DSS	DBS	Passing-Bablok	1001.564036	-4.1388	0.162591523
IL-2 Rb	DSS	DBS	Passing-Bablok	-98.79366763	2.766291	0.582715335
IL-23	DSS	DBS	Passing-Bablok	1253.560115	1.441715	0.230837605
IL-28A	DSS	DBS	Passing-Bablok	54.60341526	1.369457	0.553483587
IL-29	DSS	DBS	Passing-Bablok	-2322.059394	1.438995	0.580788571
IL-31	DSS	DBS	Passing-Bablok	-37.51109769	2.66992	0.966415801
IL-6R	DSS	DBS	Passing-Bablok	439.4335159	0.754483	0.767752623
IL-9	DSS	DBS	Passing-Bablok	451.6000023	2.038918	0.709110792
Insulin	DSS	DBS	Passing-Bablok	49.85165976	1.246688	0.831423182
IP-10	DSS	DBS	Passing-Bablok	-52.56635735	3.349141	0.38170703
I-TAC	DSS	DBS	Passing-Bablok	284.4914879	0.921281	0.139312121
LAP(TGFb1)	DSS	DBS	Passing-Bablok	378.8160153	0.492747	0.165638044
LIF	DSS	DBS	Passing-Bablok	121.6358976	1.208714	0.421230668
LIGHT	DSS	DBS	Passing-Bablok	-0.168014642	1.288486	0.363780217
LIMPII	DSS	DBS	Passing-Bablok	-51.7786207	1.514064	0.912698592
Lipocalin-2	DSS	DBS	Passing-Bablok	878.6143453	-0.29345	9.9552E-05
L-Selectin	DSS	DBS	Passing-Bablok	27216.98755	-0.73927	0.074057245
Lymphotactin	DSS	DBS	Passing-Bablok	-592.7996421	4.901775	0.181523458
LYVE-1	DSS	DBS	Passing-Bablok	147.6085821	1.114615	0.26668778
MCP-1	DSS	DBS	Passing-Bablok	0.836938149	1.002888	0.456114279
MCP-2	DSS	DBS	Passing-Bablok	-0.642872809	1.871498	0.730700807
MCP-3	DSS	DBS	Passing-Bablok	-5.478549577	4.057607	0.272328811
MCP-4	DSS	DBS	Passing-Bablok	0.67876343	2.031327	0.738476008
MCSF R	DSS	DBS	Passing-Bablok	1173.923175	0.769635	0.337670894
MDC	DSS	DBS	Passing-Bablok	89.97165932	0.554534	0.092671851
MICA	DSS	DBS	Passing-Bablok	-7.002657885	0.468404	0.901590062
MICB	DSS	DBS	Passing-Bablok	-149.4426598	1.184377	0.903105062
MIF	DSS	DBS	Passing-Bablok	2758.094476	-0.00631	-0.316757784
MIG	DSS	DBS	Passing-Bablok	-0.246300356	1.841321	0.876370394
MIP-1a	DSS	DBS	Passing-Bablok	-13.83239518	1.965696	0.93960135
MIP-1b	DSS	DBS	Passing-Bablok	-0.127508683	0.61845	0.5386882
MIP-1d	DSS	DBS	Passing-Bablok	72.82257059	1.170349	0.848864669
MIP-3a	DSS	DBS	Passing-Bablok	14.99908239	-1.10773	0.105526388
MIP-3b	DSS	DBS	Passing-Bablok	-175.5887442	3.482793	0.839475024
MPIF-1	DSS	DBS	Passing-Bablok	135.6071018	0.77984	0.635835775

Biomarker	Y	X	Regression	constant	slope	Pearson's r
4-1BB	Serum	DBS	Passing-Bablok	-58.6264	4.939851	0.917623229
6Ckine	Serum	DBS	Passing-Bablok	-4611.07	0.746606	0.422128595
Activin A	Serum	DBS	Passing-Bablok	-3.78645	0.585031	0.234240511
AgRP	Serum	DBS	Passing-Bablok	-85.7078	1.140129	0.71524924
ALCAM	Serum	DBS	Passing-Bablok	-934.778	3.957472	0.345180087
ANG-1	Serum	DBS	Passing-Bablok	-0.2759	0.422137	0.285801589
Angiogenin	Serum	DBS	Passing-Bablok	904.1534	-0.23793	0.043608992
Angiostatin	Serum	DBS	Passing-Bablok	40415.94	-0.32152	-0.427688004
AR	Serum	DBS	Passing-Bablok	13.94359	1.532694	0.673292478
Axl	Serum	DBS	Passing-Bablok	-7.00739	1.105145	0.452958814
B7-1	Serum	DBS	Passing-Bablok	-35.1459	1.420828	0.827871884
BCMA	Serum	DBS	Passing-Bablok	-3090.59	5.331388	0.486380663
BDNF	Serum	DBS	Passing-Bablok	-28.041	2.016091	0.62028746
BLC	Serum	DBS	Passing-Bablok	0.277977	1.933177	0.988801041
BMP-5	Serum	DBS	Passing-Bablok	1419.082	0.86863	0.775183993
b-NGF	Serum	DBS	Passing-Bablok	2.473055	1.637366	0.605221567
BTC	Serum	DBS	Passing-Bablok	-66.6506	1.560142	0.01240371
Cathepsin S	Serum	DBS	Passing-Bablok	-694.815	1.266912	0.806038724
CCL28	Serum	DBS	Passing-Bablok	-121.374	2.160713	0.944763124
CD14	Serum	DBS	Passing-Bablok	-454.261	1.103668	0.502818812
CD40	Serum	DBS	Passing-Bablok	-93.1453	1.40565	0.427631069
CD40L	Serum	DBS	Passing-Bablok	0.491431	0.393767	0.216607512
CEACAM-1	Serum	DBS	Passing-Bablok	-38.5673	0.654651	0.28437695
Cripto-1	Serum	DBS	Passing-Bablok	-59.42	1.687873	0.668833746
CXCL16	Serum	DBS	Passing-Bablok	-1225.58	4.562676	0.790965237
DAN	Serum	DBS	Passing-Bablok	-63.3368	0.655397	0.067961781
DKK-1	Serum	DBS	Passing-Bablok	-573.222	0.848466	0.168596672
DR6	Serum	DBS	Passing-Bablok	-1039.98	9.799269	0.589234148
Dtk	Serum	DBS	Passing-Bablok	-43.8617	3.266073	0.777541225
E-Cadherin	Serum	DBS	Passing-Bablok	-589.688	0.90901	0.139785516
EGF	Serum	DBS	Passing-Bablok	-0.1451	0.404462	0.123741736
EGF R	Serum	DBS	Passing-Bablok	-317.379	1.946171	0.641942762
EG-VEGF	Serum	DBS	Passing-Bablok	2.697819	0.871644	0.680052508
ENA-78	Serum	DBS	Passing-Bablok	30.3545	0.093903	0.480445579
Endoglin	Serum	DBS	Passing-Bablok	-64.3127	0.883847	0.270074862
Eotaxin	Serum	DBS	Passing-Bablok	11.26326	-0.64271	0.309104859
Eotaxin-2	Serum	DBS	Passing-Bablok	-4.23778	2.514991	0.648772889
Eotaxin-3	Serum	DBS	Passing-Bablok	-128.445	1.615463	0.858930568
EpCAM	Serum	DBS	Passing-Bablok	-13.0755	1.18149	0.928869532
ErbB3	Serum	DBS	Passing-Bablok	-251.728	4.466494	0.320518929
E-Selectin	Serum	DBS	Passing-Bablok	-135.428	4.877891	0.45589902
Fas	Serum	DBS	Passing-Bablok	-51.8793	2.524927	0.61060722
FAS L	Serum	DBS	Passing-Bablok	-13.6492	0.600963	0.918990591
Fcg RIIBC	Serum	DBS	Passing-Bablok	-2.26755	0.213864	0.691065728
FGF-4	Serum	DBS	Passing-Bablok	-29.9154	1.667677	0.339455965
Flt-3L	Serum	DBS	Passing-Bablok	4.163033	0.685384	0.263983944
Follistatin	Serum	DBS	Passing-Bablok	-701.315	1.68385	0.565169405
Galectin-7	Serum	DBS	Passing-Bablok	-2324.15	1.039509	0.816633033
GCP-2	Serum	DBS	Passing-Bablok	-30.7518	0.507195	0.626219126
GDF-15	Serum	DBS	Passing-Bablok	-112.409	1.870683	0.101895823
GDNF	Serum	DBS	Passing-Bablok	0	2.091446	0.819548404
GH	Serum	DBS	Passing-Bablok	16.63557	1.546124	0.637302205
GITR	Serum	DBS	Passing-Bablok	-16.1331	2.405314	0.823745289
gp130	Serum	DBS	Passing-Bablok	-3100.62	2.038721	0.654393524
HB-EGF	Serum	DBS	Passing-Bablok	-0.64122	0.855844	0.716373646
HCC-1	Serum	DBS	Passing-Bablok	3116.227	-1.22018	0.035198481
HCC-4	Serum	DBS	Passing-Bablok	-10.7611	1.456966	0.584373414
HGF	Serum	DBS	Passing-Bablok	-28.4737	1.844944	0.623004954
HVEM	Serum	DBS	Passing-Bablok	-179.802	2.143494	0.702095469
ICAM-1	Serum	DBS	Passing-Bablok	-2223.04	2.962729	0.75229428
ICAM-2	Serum	DBS	Passing-Bablok	-2302.07	1.659733	0.816542079
ICAM-3	Serum	DBS	Passing-Bablok	-27.1393	1.08453	0.710780532
IGFBP-1	Serum	DBS	Passing-Bablok	-64.674	1.0757	0.605865854
IGFBP-2	Serum	DBS	Passing-Bablok	-105.903	1.52363	0.469869573
IGFBP-3	Serum	DBS	Passing-Bablok	523.5781	0.172486	0.371712248
IGFBP-4	Serum	DBS	Passing-Bablok	-21511.6	2.091599	0.419186151
IGFBP-6	Serum	DBS	Passing-Bablok	1376.643	0.519123	0.122349194
IL-1 R4	Serum	DBS	Passing-Bablok	2.115993	1.453484	0.657152272
IL-1 RI	Serum	DBS	Passing-Bablok	-0.52814	1.537291	0.693396685
IL-10 Rb	Serum	DBS	Passing-Bablok	-37.4991	2.30099	0.914784681
IL-12p40	Serum	DBS	Passing-Bablok	-7.85871	4.70741	0.586273389
IL-13 R1	Serum	DBS	Passing-Bablok	-876.839	1.836974	0.685713253
IL-13 R2	Serum	DBS	Passing-Bablok	-73.4567	2.138562	0.802741453
IL-15	Serum	DBS	Passing-Bablok	6.511517	1.188535	0.971636584
IL-16	Serum	DBS	Passing-Bablok	-123.967	1.050084	0.901137696
IL-17B	Serum	DBS	Passing-Bablok	-1944.45	2.320436	0.545564976
IL-17F	Serum	DBS	Passing-Bablok	-1394.59	2.282907	0.879607109
IL-17R	Serum	DBS	Passing-Bablok	-1358.2	2.402652	0.729013536
IL-18 BPa	Serum	DBS	Passing-Bablok	-1655.78	1.244432	0.534408477
IL-2 Ra	Serum	DBS	Passing-Bablok	-136.08	1.750099	0.367193225
IL-2 Rb	Serum	DBS	Passing-Bablok	-2191.55	2.031992	0.444770442
IL-21R	Serum	DBS	Passing-Bablok	-561.016	2.041918	0.81429114
IL-23	Serum	DBS	Passing-Bablok	-1416.6	1.157795	0.823632169
IL-28A	Serum	DBS	Passing-Bablok	-3.65998	0.3287	0.62664942
IL-31	Serum	DBS	Passing-Bablok	-104.643	2.874496	0.927968658
IL-6R	Serum	DBS	Passing-Bablok	245.9927	0.97467	0.53236218
Insulin	Serum	DBS	Passing-Bablok	16.94926	1.663006	0.881114454
IP-10	Serum	DBS	Passing-Bablok	-20.3388	1.229038	0.448228705
LAP(TGFb1)	Serum	DBS	Passing-Bablok	18.75412	0.2367	0.164182156
LIGHT	Serum	DBS	Passing-Bablok	-70.3327	0.540767	0.06587461
LIMPII	Serum	DBS	Passing-Bablok	-205.927	6.294792	0.939207799
Lipocalin-2	Serum	DBS	Passing-Bablok	469.3908	-0.1893	-0.22960383
L-Selectin	Serum	DBS	Passing-Bablok	-20327.8	3.125197	0.181954819
Lymphotactin	Serum	DBS	Passing-Bablok	-258.485	1.509824	0.671385353
LYVE-1	Serum	DBS	Passing-Bablok	-376.83	1.918207	0.387719515
MCP-2	Serum	DBS	Passing-Bablok	-9.00634	0.64614	0.721407367
MCP-3	Serum	DBS	Passing-Bablok	-0.77428	0.691095	0.305310975
MCP-4	Serum	DBS	Passing-Bablok	-1.16041	1.510981	0.543781462
MCSF R	Serum	DBS	Passing-Bablok	1393.306	0.888239	0.457538068
MDC	Serum	DBS	Passing-Bablok	27.36266	0.11691	0.240243863
MICA	Serum	DBS	Passing-Bablok	-7.74478	0.835371	0.73694329
MICB	Serum	DBS	Passing-Bablok	-442.025	1.930924	0.899355758
MIF	Serum	DBS	Passing-Bablok	524.4205	-0.00569	-0.479476906
MIG	Serum	DBS	Passing-Bablok	-0.04838	2.46288	0.774710657
MIP-1a	Serum	DBS	Passing-Bablok	-22.311	2.978616	0.863302733
MIP-1d	Serum	DBS	Passing-Bablok	-13.8992	2.764244	0.595676881
MIP-3a	Serum	DBS	Passing-Bablok	-0.51147	0.285833	0.289823149
MIP-3b	Serum	DBS	Passing-Bablok	-441.194	5.494564	0.738691151
MPIF-1	Serum	DBS	Passing-Bablok	-15.0446	1.264132	0.21638435
MSP	Serum	DBS	Passing-Bablok	-1008.72	5.070694	0.524852962
NAP-2	Serum	DBS	Passing-Bablok	105.9586	0.606888	0.304999859
NGF R	Serum	DBS	Passing-Bablok	4.52706	1.284885	0.702428099
NrCAM	Serum	DBS	Passing-Bablok	-127.468	0.655282	0.521241123
NT-3	Serum	DBS	Passing-Bablok	4.057967	1.404976	0.771422645
NT-4	Serum	DBS	Passing-Bablok	7.517967	1.438102	0.612367356
OPG	Serum	DBS	Passing-Bablok	0.494454	0.268651	0.445815553
OPN	Serum	DBS	Passing-Bablok	246.9517	0.043112	0.206306188

Biomarker	Y	X	Regression	constant	slope	Pearson's r
4-1BB	Serum	DSS	Passing-Bablok	2.261484	2.561234	0.972403858
6Ckine	Serum	DSS	Passing-Bablok	345.2986	0.242004	0.366077692
Activin A	Serum	DSS	Passing-Bablok	311.1393	-0.46854	0.070104372
AgRP	Serum	DSS	Passing-Bablok	-7.52614	0.275216	0.223072395
ALCAM	Serum	DSS	Passing-Bablok	-297.044	1.843205	0.358406507
ANG-1	Serum	DSS	Passing-Bablok	24.70453	0.423667	0.687310671
Angiogenin	Serum	DSS	Passing-Bablok	-532.089	1.836146	0.088151089
Angiostatin	Serum	DSS	Passing-Bablok	-11786.6	0.486574	0.220557883
AR	Serum	DSS	Passing-Bablok	-5.08341	1.154855	0.802287478
Axl	Serum	DSS	Passing-Bablok	-5.66007	0.601755	0.550095412
B7-1	Serum	DSS	Passing-Bablok	-7.56399	1.243984	0.913072625
BCMA	Serum	DSS	Passing-Bablok	-1089.96	2.402296	0.688438723
BDNF	Serum	DSS	Passing-Bablok	-15.7183	2.388938	0.867656649
bFGF	Serum	DSS	Passing-Bablok	-5.42744	0.661718	0.377647718
BLC	Serum	DSS	Passing-Bablok	0.210159	1.436657	0.990784185
BMP-5	Serum	DSS	Passing-Bablok	-10581.9	1.26013	0.570923321
BMP-7	Serum	DSS	Passing-Bablok	0	1.66843	0.893149153
b-NGF	Serum	DSS	Passing-Bablok	-1.65587	1.23016	0.708815562
BTC	Serum	DSS	Passing-Bablok	-20.6484	0.824093	0.534272254
Cathepsin S	Serum	DSS	Passing-Bablok	-976.513	1.748329	0.928855858
CCL28	Serum	DSS	Passing-Bablok	-421.579	1.417512	0.977910823
CD14	Serum	DSS	Passing-Bablok	-3151.38	1.684165	0.349528118
CD40	Serum	DSS	Passing-Bablok	-41.4373	0.449189	0.579421489
CD40L	Serum	DSS	Passing-Bablok	-1.04901	0.429735	0.1706505
CEACAM-1	Serum	DSS	Passing-Bablok	-35.1417	0.524748	0.318500627
Cripto-1	Serum	DSS	Passing-Bablok	-69.6759	1.515872	0.801794664
CXCL16	Serum	DSS	Passing-Bablok	-1059.47	2.345103	0.839105328
DAN	Serum	DSS	Passing-Bablok	-87.4532	0.343695	0.328451109
DKK-1	Serum	DSS	Passing-Bablok	-741.377	0.671434	0.549871697
DR6	Serum	DSS	Passing-Bablok	-609.022	5.280602	0.814800881
Dtk	Serum	DSS	Passing-Bablok	-13.5201	2.01968	0.87834955
E-Cadherin	Serum	DSS	Passing-Bablok	-914.091	0.978918	0.665038951
EGF	Serum	DSS	Passing-Bablok	0	1.476577	0.843595337
EGF R	Serum	DSS	Passing-Bablok	65.08508	1.501823	0.909876936
EG-VEGF	Serum	DSS	Passing-Bablok	-1.97912	1.205052	0.770713828
ENA-78	Serum	DSS	Passing-Bablok	-68.0635	0.851599	0.78871576
Endoglin	Serum	DSS	Passing-Bablok	4.068075	1.545489	0.580086161
Eotaxin	Serum	DSS	Passing-Bablok	-1.82792	3.204933	0.767047217
Eotaxin-2	Serum	DSS	Passing-Bablok	-10.5944	3.712849	0.626795623
Eotaxin-3	Serum	DSS	Passing-Bablok	-228.867	0.671825	0.840656977
EpCAM	Serum	DSS	Passing-Bablok	-30.3931	1.01756	0.945038381
ErbB3	Serum	DSS	Passing-Bablok	-45.5074	1.77034	0.380776996
E-Selectin	Serum	DSS	Passing-Bablok	-589.124	10.23026	0.006994614
Fas	Serum	DSS	Passing-Bablok	-35.39	3.612895	0.641467647
FAS L	Serum	DSS	Passing-Bablok	-28.3529	0.604302	0.894773965
Fcg RIIBC	Serum	DSS	Passing-Bablok	17.24714	0.113427	0.650707186
FGF-4	Serum	DSS	Passing-Bablok	0	1.32649	0.641563766
FGF-7	Serum	DSS	Passing-Bablok	0	2.037372	0.896657269
Flt-3L	Serum	DSS	Passing-Bablok	4.856509	0.818876	0.487887835
Follistatin	Serum	DSS	Passing-Bablok	-534.228	0.587661	0.617218266
Galectin-7	Serum	DSS	Passing-Bablok	-881.637	1.038779	0.943753444
GCP-2	Serum	DSS	Passing-Bablok	-31.3135	0.526749	0.659281328
GDF-15	Serum	DSS	Passing-Bablok	-72.1653	1.248932	0.251155166
GDNF	Serum	DSS	Passing-Bablok	-8.57749	1.836651	0.903461677
GH	Serum	DSS	Passing-Bablok	2.94793	1.440347	0.866439284
gp130	Serum	DSS	Passing-Bablok	-1171.22	0.935041	0.791382144
HB-EGF	Serum	DSS	Passing-Bablok	-0.52117	0.770532	0.943665277
HCC-1	Serum	DSS	Passing-Bablok	-167.633	0.895715	0.066237495
HCC-4	Serum	DSS	Passing-Bablok	142.862	0.811865	0.351113184
HGF	Serum	DSS	Passing-Bablok	-17.0632	1.988586	0.771917648
HVEM	Serum	DSS	Passing-Bablok	-36.8404	1.502308	0.735148276
ICAM-1	Serum	DSS	Passing-Bablok	-1148.6	2.102853	0.864365435
ICAM-2	Serum	DSS	Passing-Bablok	-5587.82	1.534805	0.844228292
ICAM-3	Serum	DSS	Passing-Bablok	0	0.964882	0.739373234
IGF-1	Serum	DSS	Passing-Bablok	0	2.68449	0.918455534
IGFBP-1	Serum	DSS	Passing-Bablok	12.80423	1.428346	0.931780973
IGFBP-2	Serum	DSS	Passing-Bablok	1407.246	1.19804	0.346669511
IGFBP-3	Serum	DSS	Passing-Bablok	-16.8626	0.166311	0.566832934
IGFBP-4	Serum	DSS	Passing-Bablok	-10569.9	0.713534	0.671719228
IGFBP-6	Serum	DSS	Passing-Bablok	440.0799	0.416503	0.195630869
IL-1 R4	Serum	DSS	Passing-Bablok	-11.7218	1.338607	0.6918677
IL-1 RI	Serum	DSS	Passing-Bablok	-0.01343	1.779551	0.788166687
IL-10 Rb	Serum	DSS	Passing-Bablok	-5.36799	1.873997	0.941217765
IL-11	Serum	DSS	Passing-Bablok	0	0.423586	0.855644259
IL-12p40	Serum	DSS	Passing-Bablok	-5.35804	3.424301	0.632203053
IL-13 R1	Serum	DSS	Passing-Bablok	-685.215	1.023592	0.761306851
IL-13 R2	Serum	DSS	Passing-Bablok	-238.046	1.319178	0.877775626
IL-15	Serum	DSS	Passing-Bablok	-2.66066	1.156764	0.956333476
IL-16	Serum	DSS	Passing-Bablok	0.246436	0.91516	0.972635232
IL-17B	Serum	DSS	Passing-Bablok	-907.065	0.625757	0.473746267
IL-17F	Serum	DSS	Passing-Bablok	-482.1	1.142326	0.923633372
IL-17R	Serum	DSS	Passing-Bablok	-432.539	1.577276	0.815219109
IL-18 BPa	Serum	DSS	Passing-Bablok	-659.595	0.740039	0.78381844
IL-2 Ra	Serum	DSS	Passing-Bablok	-131.862	0.664421	0.51604888
IL-2 Rb	Serum	DSS	Passing-Bablok	-1599.61	0.654173	0.498032802
IL-23	Serum	DSS	Passing-Bablok	-2748.52	0.860577	0.145900404
IL-28A	Serum	DSS	Passing-Bablok	-29.3528	0.282134	0.42332706
IL-31	Serum	DSS	Passing-Bablok	-61.0761	1.179306	0.952642986
IL-6R	Serum	DSS	Passing-Bablok	-314.72	1.337211	0.659885291
Insulin	Serum	DSS	Passing-Bablok	-71.2631	1.892379	0.819459196
IP-10	Serum	DSS	Passing-Bablok	-2.8642	0.417851	0.414314508
LAP(TGFb1)	Serum	DSS	Passing-Bablok	-29.3418	0.328857	0.110654318
LIGHT	Serum	DSS	Passing-Bablok	-155.75	0.719826	0.79304632
LIMPII	Serum	DSS	Passing-Bablok	-16.5448	3.098188	0.919456528
Lipocalin-2	Serum	DSS	Passing-Bablok	-30.9631	0.430503	0.007961264
L-Selectin	Serum	DSS	Passing-Bablok	-46247.8	4.19626	0.160555866
Lymphotactin	Serum	DSS	Passing-Bablok	-112.958	0.371464	0.470244711
LYVE-1	Serum	DSS	Passing-Bablok	-413.596	1.548042	0.507539621
MCP-2	Serum	DSS	Passing-Bablok	-13.45	0.38914	0.946866968
MCP-3	Serum	DSS	Passing-Bablok	-0.49262	0.17667	0.148541262
MCP-4	Serum	DSS	Passing-Bablok	-1.65341	0.658436	0.669095868
MCSF R	Serum	DSS	Passing-Bablok	-341.705	1.243417	0.388743407
MDC	Serum	DSS	Passing-Bablok	5.126682	0.220676	0.642868046
MICA	Serum	DSS	Passing-Bablok	10.87659	2.545797	0.850043458
MICB	Serum	DSS	Passing-Bablok	-121.127	1.404591	0.773971039
MIF	Serum	DSS	Passing-Bablok	-88.4299	0.167316	0.653930875
MIG	Serum	DSS	Passing-Bablok	5.905022	1.418681	0.923474859
MIP-1a	Serum	DSS	Passing-Bablok	-0.52718	1.581758	0.687455171
MIP-1d	Serum	DSS	Passing-Bablok	-187.114	2.350364	0.753970458
MIP-3a	Serum	DSS	Passing-Bablok	-1.04274	0.215597	0.541463357
MIP-3b	Serum	DSS	Passing-Bablok	-100.03	1.471975	0.890743898
MPIF-1	Serum	DSS	Passing-Bablok	-144.806	1.60966	0.280956589
MSP	Serum	DSS	Passing-Bablok	-1287.14	4.381347	0.648731042
NAP-2	Serum	DSS	Passing-Bablok	-88.0406	1.347318	0.384366944
NGF R	Serum	DSS	Passing-Bablok	2.937097	1.38948	0.614038899
NrCAM	Serum	DSS	Passing-Bablok	-91.6809	0.3693	0.631168037
NT-3	Serum	DSS	Passing-Bablok	-23.0492	1.674273	0.554807034

MSP	DSS	DBS	Passing-Bablok	-166.7412799	1.5052	0.26915111
NAP-2	DSS	DBS	Passing-Bablok	157.6189001	0.456963	-0.005206804
NGF R	DSS	DBS	Passing-Bablok	1.89135527	0.988597	0.720664906
NrCAM	DSS	DBS	Passing-Bablok	-86.61976905	1.70985	0.70308586
NT-3	DSS	DBS	Passing-Bablok	8.356764397	0.874336	0.826065979
NT-4	DSS	DBS	Passing-Bablok	13.18794769	1.313446	0.775637247
OPG	DSS	DBS	Passing-Bablok	3.652235361	0.740324	0.172485248
OPN	DSS	DBS	Passing-Bablok	720.0235284	1.202291	0.217637239
PAI-1	DSS	DBS	Passing-Bablok	520.704029	0.876539	0.5124436
PARC	DSS	DBS	Passing-Bablok	1.200379686	1.092502	0.234263572
PDGF Rb	DSS	DBS	Passing-Bablok	0	1.577756	0.830697538
PDGF-AA	DSS	DBS	Passing-Bablok	-217.9639387	1.187783	0.283104709
PDGF-AB	DSS	DBS	Passing-Bablok	-144.1682003	1.229226	0.401955296
PDGF-BB	DSS	DBS	Passing-Bablok	93.5532503	0.801982	0.374163515
PECAM-1	DSS	DBS	Passing-Bablok	798.988756	-0.28383	0.007201684
PF4	DSS	DBS	Passing-Bablok	-2210.831071	1.178105	0.048611834
PIGF	DSS	DBS	Passing-Bablok	0.757830057	0.986091	0.808555216
RAGE	DSS	DBS	Passing-Bablok	-47.67269305	1.116414	0.706614376
RANTES	DSS	DBS	Passing-Bablok	-2139.295852	1.669902	0.377606652
Resistin	DSS	DBS	Passing-Bablok	64.73445865	0.083828	0.26422396
SCF	DSS	DBS	Passing-Bablok	2.031665041	1.087851	0.740709896
SCF R	DSS	DBS	Passing-Bablok	-2161.012565	2.353951	0.151355288
SDF-1a	DSS	DBS	Passing-Bablok	-16.96229371	2.658189	0.535281233
SDF-1b	DSS	DBS	Passing-Bablok	-28.33554658	1.420447	0.799782838
Shh-N	DSS	DBS	Passing-Bablok	-18.63076437	2.125917	0.663902062
Siglec-5	DSS	DBS	Passing-Bablok	-63.58560594	1.245819	0.724593889
TARC	DSS	DBS	Passing-Bablok	7.261916885	0.968741	0.624830738
TECK	DSS	DBS	Passing-Bablok	-12.5693413	2.886489	0.60134809
TGFa	DSS	DBS	Passing-Bablok	-3.809852312	1.464013	0.933937509
TGFb1	DSS	DBS	Passing-Bablok	8846.898259	1.341563	0.807925787
TGFb2	DSS	DBS	Passing-Bablok	-422.2724824	2.642464	0.345693838
TGFb3	DSS	DBS	Passing-Bablok	4.142342842	1.988645	0.716430063
Tie-2	DSS	DBS	Passing-Bablok	606.2977034	1.309566	0.416083008
TIM-1	DSS	DBS	Passing-Bablok	-19.2348396	1.683591	0.971834484
TIMP-1	DSS	DBS	Passing-Bablok	656.6054572	0.814529	0.730364895
TIMP-2	DSS	DBS	Passing-Bablok	-104.1561021	1.02631	0.094257986
TNF RI	DSS	DBS	Passing-Bablok	-91.21707947	2.247315	0.205876444
TNF RII	DSS	DBS	Passing-Bablok	-1703.406326	3.278873	0.28789033
TPO	DSS	DBS	Passing-Bablok	-1333.407446	3.365937	0.421463216
TRAIL R3	DSS	DBS	Passing-Bablok	2.758251475	0.602985	0.40023998
TRAIL R4	DSS	DBS	Passing-Bablok	-97.06313892	3.470983	0.387046547
Trappin-2	DSS	DBS	Passing-Bablok	555.2054128	0.509766	0.661017832
TREM-1	DSS	DBS	Passing-Bablok	454.7542243	2.002745	0.31796767
TSLP	DSS	DBS	Passing-Bablok	-49.01837625	2.361953	0.954829041
uPAR	DSS	DBS	Passing-Bablok	31.35890149	0.21482	0.412691351
VCAM-1	DSS	DBS	Passing-Bablok	-871.7012459	1.695549	0.63202299
VEGF	DSS	DBS	Passing-Bablok	-0.439550641	0.922613	0.740202181
VEGF R1	DSS	DBS	Passing-Bablok	49.91489096	1.879555	0.805259192
VEGF R2	DSS	DBS	Passing-Bablok	-14.15538092	1.70246	0.510882869
VEGF R3	DSS	DBS	Passing-Bablok	16.20762054	1.281921	0.681229383
VEGF-C	DSS	DBS	Passing-Bablok	-83.00698591	1.858412	0.356028873
VEGF-D	DSS	DBS	Passing-Bablok	0.122723441	1.690206	0.754239191

PAI-1	Serum	DBS	Passing-Bablok	147.6874	0.823051	0.36736086
PARC	Serum	DBS	Passing-Bablok	8.410148	0.50861	0.531002124
PDGF Rb	Serum	DBS	Passing-Bablok	0	1.464825	0.548650188
PDGF-AA	Serum	DBS	Passing-Bablok	-459.002	2.093589	0.212872871
PDGF-AB	Serum	DBS	Passing-Bablok	-490.865	1.144093	0.244696477
PDGF-BB	Serum	DBS	Passing-Bablok	-1424.18	1.953097	0.316099402
PECAM-1	Serum	DBS	Passing-Bablok	627.2544	-0.26423	-0.115087487
PF4	Serum	DBS	Passing-Bablok	2977.825	0.773121	0.20900244
PIGF	Serum	DBS	Passing-Bablok	0.609177	0.609462	0.76076554
RAGE	Serum	DBS	Passing-Bablok	-151.037	1.847138	0.360059347
RANTES	Serum	DBS	Passing-Bablok	-2220.07	1.701275	0.492351109
Resistin	Serum	DBS	Passing-Bablok	-215.401	0.157254	0.353004248
SCF	Serum	DBS	Passing-Bablok	-3.26435	0.633325	0.593164935
SCF R	Serum	DBS	Passing-Bablok	-3310.41	3.247103	0.351541388
SDF-1a	Serum	DBS	Passing-Bablok	-79.9495	1.527633	0.656183765
SDF-1b	Serum	DBS	Passing-Bablok	-90.6902	1.326642	0.83346823
Shh-N	Serum	DBS	Passing-Bablok	-65.849	1.506592	0.428690064
Siglec-5	Serum	DBS	Passing-Bablok	-1358.29	3.420696	0.121891535
TARC	Serum	DBS	Passing-Bablok	-2.15346	0.576895	0.265187519
TECK	Serum	DBS	Passing-Bablok	-11.676	0.588471	0.382070475
TGFa	Serum	DBS	Passing-Bablok	-4.06396	2.038018	0.741369856
TGFb1	Serum	DBS	Passing-Bablok	-2265.55	0.899544	0.711313569
TGFb2	Serum	DBS	Passing-Bablok	-730.66	1.564455	0.540101291
Tie-2	Serum	DBS	Passing-Bablok	-145.927	0.680448	0.512866613
TIM-1	Serum	DBS	Passing-Bablok	-66.8441	5.322545	0.935971465
TIMP-1	Serum	DBS	Passing-Bablok	-1330.23	1.172169	0.35985287
TIMP-2	Serum	DBS	Passing-Bablok	7701.088	-1.63283	-0.045947617
TNF RI	Serum	DBS	Passing-Bablok	2895.908	-19.9925	-0.102201872
TNF RII	Serum	DBS	Passing-Bablok	-4215.98	6.623214	0.273370164
TPO	Serum	DBS	Passing-Bablok	-1080.19	1.371183	0.312836805
TRAIL R3	Serum	DBS	Passing-Bablok	-51.4744	0.659474	0.199051135
TRAIL R4	Serum	DBS	Passing-Bablok	-72.5578	1.45984	0.379810126
Trappin-2	Serum	DBS	Passing-Bablok	-227.131	1.418545	0.258832962
TREM-1	Serum	DBS	Passing-Bablok	-136.001	0.445554	0.557289434
TSLP	Serum	DBS	Passing-Bablok	-73.4319	2.216239	0.652048943
uPAR	Serum	DBS	Passing-Bablok	-114.963	0.586104	0.230774389
VCAM-1	Serum	DBS	Passing-Bablok	-33325.5	5.212885	0.280663461
VEGF R1	Serum	DBS	Passing-Bablok	-447.427	1.636683	0.849048294
VEGF R2	Serum	DBS	Passing-Bablok	-124.789	1.753424	0.634005688
VEGF R3	Serum	DBS	Passing-Bablok	308.4218	1.708432	0.699558045
VEGF-C	Serum	DBS	Passing-Bablok	-95.9509	0.590343	0.266890399
VEGF-D	Serum	DBS	Passing-Bablok	36.8476	3.599315	0.760814217

NT-4	Serum	DSS	Passing-Bablok	3.146957	1.024301	0.892853046
OPG	Serum	DSS	Passing-Bablok	0.210793	0.369507	-0.020118053
OPN	Serum	DSS	Passing-Bablok	-51.6301	0.051451	0.222458385
PAI-1	Serum	DSS	Passing-Bablok	201.6262	0.827512	0.326875773
PARC	Serum	DSS	Passing-Bablok	15.52166	0.309275	0.212747258
PDGF Rb	Serum	DSS	Passing-Bablok	0	0.801644	0.772600667
PDGF-AA	Serum	DSS	Passing-Bablok	4.88698	1.465036	0.809943951
PDGF-AB	Serum	DSS	Passing-Bablok	-489.847	1.141695	0.623491242
PDGF-BB	Serum	DSS	Passing-Bablok	-931.401	1.908286	0.752087897
PECAM-1	Serum	DSS	Passing-Bablok	-30.9612	0.701506	0.891610755
PF4	Serum	DSS	Passing-Bablok	35966.71	-0.57211	-0.014114335
PIGF	Serum	DSS	Passing-Bablok	0.128805	0.687135	0.54359814
RAGE	Serum	DSS	Passing-Bablok	-94.0204	1.69203	0.191459795
RANTES	Serum	DSS	Passing-Bablok	204.2868	0.921619	0.793805343
Resistin	Serum	DSS	Passing-Bablok	-68.6339	1.031725	0.278669761
SCF	Serum	DSS	Passing-Bablok	-2.96381	0.469388	0.450046546
SCF R	Serum	DSS	Passing-Bablok	-378.703	1.431627	0.606700413
SDF-1a	Serum	DSS	Passing-Bablok	-71.648	0.440952	0.614791435
SDF-1b	Serum	DSS	Passing-Bablok	-93.544	0.885678	0.834345709
Shh-N	Serum	DSS	Passing-Bablok	-57.1796	0.605113	0.63089405
Siglec-5	Serum	DSS	Passing-Bablok	-1137.01	2.855528	0.392974372
TARC	Serum	DSS	Passing-Bablok	-4.00583	0.530948	0.365420149
TECK	Serum	DSS	Passing-Bablok	-22.8419	0.21094	0.385429689
TGFa	Serum	DSS	Passing-Bablok	4.042202	1.383438	0.835597474
TGFb1	Serum	DSS	Passing-Bablok	-10334.2	0.871666	0.597025997
TGFb2	Serum	DSS	Passing-Bablok	-784.28	0.562707	0.500551416
Tie-2	Serum	DSS	Passing-Bablok	-350.033	0.460367	0.600097739
TIM-1	Serum	DSS	Passing-Bablok	1.817291	2.698174	0.94940627
TIMP-1	Serum	DSS	Passing-Bablok	-2467.23	1.618775	0.420093922
TIMP-2	Serum	DSS	Passing-Bablok	-411.568	1.365311	0.47128447
TNF RI	Serum	DSS	Passing-Bablok	-500.582	5.192158	0.194515037
TNF RII	Serum	DSS	Passing-Bablok	-1119.89	2.507857	0.420952303
TPO	Serum	DSS	Passing-Bablok	-429.761	0.406159	0.654054838
TRAIL R3	Serum	DSS	Passing-Bablok	-69.314	1.055803	0.510203858
TRAIL R4	Serum	DSS	Passing-Bablok	-24.9768	0.541209	0.62733488
Trappin-2	Serum	DSS	Passing-Bablok	-1660.29	2.884783	0.440979372
TREM-1	Serum	DSS	Passing-Bablok	-285.052	0.254331	0.422842932
TSLP	Serum	DSS	Passing-Bablok	-35.7229	1.116915	0.817377016
uPAR	Serum	DSS	Passing-Bablok	-98.6423	1.559711	0.642747365
VCAM-1	Serum	DSS	Passing-Bablok	-40866.1	4.225186	0.266203655
VEGF R1	Serum	DSS	Passing-Bablok	-960.61	0.943248	0.890710486
VEGF R2	Serum	DSS	Passing-Bablok	-152.916	1.180976	0.577814988
VEGF R3	Serum	DSS	Passing-Bablok	-132.751	1.802338	0.838203232
VEGF-C	Serum	DSS	Passing-Bablok	-114.535	0.355522	0.720518349
VEGF-D	Serum	DSS	Passing-Bablok	-13.0955	2.426605	0.922478159
XEDAR	Serum	DSS	Passing-Bablok	8.37665	7.874956	0.874144963

**Protein Target**	**Asante 60 mm^2 strips (%CV)**	**Asante 100 mm^2 strips (%CV)**	**Whatman 903 50mm^2 strips (%CV)**
Angiotensinogen	12%	12%	11%
ANGPTL3	24%	11%	16%
CHI3L1	18%	16%	13%
Dkk-3	20%	12%	11%
GASP-1	34%	16%	11%
ICAM-1	18%	11%	15%
IL-1 R3	16%	8%	9%
IL-6R	12%	7%	7%
LAP	21%	20%	17%
NAP-2	23%	18%	22%
NOV	12%	18%	13%
PARC	14%	12%	2%
P-Cadherin	17%	11%	12%
PDGF-AA	19%	26%	2%
PDGF-BB	30%	24%	10%
PGRP-s	10%	12%	14%
RANTES	24%	9%	6%
SCF R	24%	31%	7%
Siglec-5	12%	17%	20%
TIMP-1	17%	11%	9%
TIMP-2	15%	22%	9%
Trappin-2	26%	28%	21%
**Average**	19%	16%	12%

Table S6: Comparison of %CV in protein expression across 4 samples tested in a variety of filter papers and sizes.
